# Risk-period-cohort approach for averting identification problems in longitudinal models

**DOI:** 10.1371/journal.pone.0219399

**Published:** 2019-07-10

**Authors:** Douglas D. Gunzler, Adam T. Perzynski, Neal V. Dawson, Kelley Kauffman, Jintao Liu, Jarrod E. Dalton

**Affiliations:** 1 Case Western Reserve University, Center for Health Care Research & Policy, MetroHealth Medical Center, Cleveland, Ohio, United States of America; 2 Case Western Reserve University, Department of Population and Quantitative Health Sciences, Cleveland, Ohio, United States of America; 3 Case Western Reserve University, Center for Reducing Health Disparities, MetroHealth Medical Center, Cleveland, Ohio, United States of America; 4 Quantitative Health Sciences, Cleveland Clinic, Cleveland, Ohio, United States of America; Tongji University, CHINA

## Abstract

In epidemiology, gerontology, human development and the social sciences, age-period-cohort (APC) models are used to study the variability in trajectories of change over time. A well-known issue exists in simultaneously identifying age, period and birth cohort effects, namely that the three characteristics comprise a perfectly collinear system. That is, since *age* = *period*−*cohort*, only two of these effects are estimable at a time.

In this paper, we introduce an alternative framework for considering effects relating to age, period and birth cohort. In particular, instead of directly modeling age in the presence of period and cohort effects, we propose a risk modeling approach to characterize age-related risk (i.e., a hybrid of multiple biological and sociological influences to evaluate phenomena associated with growing older). The properties of this approach, termed **risk-period-cohort (RPC)**, are described in this paper and studied by simulations. We show that, except for pathological circumstances where risk is uniquely determined by age, using such risk indices obviates the problem of collinearity. We also show that the size of the chronological age effect in the risk prediction model associates with the correlation between a risk index and chronological age and that the RPC approach can satisfactorily recover cohort and period effects in most cases. We illustrate the advantages of RPC compared to traditional APC analysis on 27496 individuals from NHANES survey data (2005–2016) to study the longitudinal variability in depression screening over time. Our RPC method has broad implications for examining processes of change over time in longitudinal studies.

## Introduction

*Age-Period-Cohort* (APC) models are valuable for understanding phenomena that account for variability over time in outcome trajectories. The purpose of APC models is primarily to decompose data trends into age, period and cohort effects. *Age effects* have been described as phenomena associated with growing older; *period effects* as general influences that vary through time or epochs; and *cohort effects* as phenomena associated with individuals born around the same time (i.e., birth cohorts). APC models have been adopted in epidemiology to study a wide variety of health outcomes, including breast cancer incidence [1], trends in blood pressure and body mass index [2] and suicides [3]. APC models have been developed for individual level data using hierarchical modeling approaches and time-to-event outcomes [4, 5]. APC effects have also been shown to differ by sex and race [4, 6], for example in rates of preterm delivery [7].

While many longitudinal studies have included age and period variables, APC studies have demonstrated the advantages of evaluating differential cohort effects [4]. The influence of historic events varies depending on the stage of life at which they are experienced; one can examine the influence of such historic events on subpopulations at different ages by following cohorts over time [8]. For example, infectious disease epidemics of the 20^th^ Century (e.g. hepatitis C and human immunodeficiency viruses) influenced lives and outcomes differently for distinct birth cohorts. Similarly, technological and therapeutic innovations can have drastically different influences on the lives of different cohorts in different epochs.

A major impediment to APC research, called the “identification problem”, exists in that chronological age, period, and cohort effects are linearly dependent, since *age = period—cohort* [4, 9]. In other words, given any two of these three characteristics one can calculate the third. This identity in practice can be further clarified for this paper. Reference in the “identification problem” is to single measures for chronological age, a given time period and birth cohort to represent age, period and cohort, respectively.

Several solutions have been proposed to address the identifiability problem in the APC literature. Yang and Land (2013) [4] proposed the Hierarchical APC (HAPC) model to resolve identifiability in APC using repeated measures data across individuals. The proposed mixed effects modeling approach described in Yang and Land (2013) [4] includes fixed effects for linear and quadratic age and random effects for cohort and period.

In another proposed solution, constrained regression analysis, certain levels of categorical age, period and/or cohort factors are assumed to have the same effect on the outcome [10]. The “intrinsic estimator” method also works by constraining parameters [4]. In the “APC-characteristic” model, proxy variables (e.g., cohort size and/or percentage of cohort born out of wedlock as opposed to individual cohorts) are substituted for at least one of the three variables [8]. Nonlinear transformation of one of the APC variables has also been proposed as a solution for identifiability [4].

These proposed methods to address the “identifiability problem” rely on consequential assumptions and require researchers to impose implicit constraints on the model parameters that may fail to recover the underlying APC effects. Recent studies have shown these methods can be sensitive to different model parameterizations [9, 11–15].

Further, prior frameworks for APC analysis assume that all individuals at a given age have the same risk of outcome (say, mortality) when holding period and cohort fixed. However, aging is a process that does not happen uniformly, linearly, or sometimes even monotonically, over time. Health behaviors; functional status; comorbid illnesses; genetic and epigenetic markers; environmental and neighborhood-level exposures; medication use and other effects may introduce substantial heterogeneity in outcome risk for persons of a given chronological age. Estimates of a”biological age” have been shown to be a better predictor of mortality than chronological age [16].

We thus propose a practical approach that does not attempt to solve the identification problem, but rather employs a measure of *age-related risk* in place of chronological age. Age-related risk is the accumulation of physiological, lifestyle, environmental, sociological and other contextualizing risks as a representation of how the process of aging is affecting an individual [17, 18]. Our approach was inspired by internal risk modeling techniques used for evaluating heterogeneous treatment effects in randomized trials [19–22]. In the APC context, this new framework obviates the identifiability problem by substituting the multidimensional risk index in place of chronological age. We refer to the method as the **risk-period-cohort (RPC) approach**.

Implementing the RPC model involves using a multidimensional age-related risk index that combines chronological age and other risk factors (e.g., via multiple regression or a machine learning model) to describe the aging process with respect to an outcome of interest. Assuming three basic assumptions are met (discussed in section 2), the risk index will not be collinear with period or cohort, due to the inherent variability in the set of underlying risk factors. In theory, any suggested model for APC analysis that evaluates age-related risk along with period and cohort effects can be used with this technique.

In brief, we are proposing an alternative qualitative framework for APC analysis; the existing approaches use a framework which includes an identification problem. The major advantage of the risk-period-cohort (RPC) approach over all existing approaches is that (a) it does not have an identification problem and (b) it is able to better capture phenomena related to processes of change over the life course.

For illustration in outlining the RPC modeling approach, in section 2, we describe modifications to extend the Yang and Land’s HAPC framework [4] for our RPC approach for APC analysis. In section 3 we describe simulation studies to evaluate the (1) relationship between the size of the chronological age effect in the risk model and the correlation between age-related risk index and chronological age and (2) proficiency of the risk modeling approach, given a correctly specified internal risk model, in recovering additional cohort and period effects. In section 4 we use a health data example to illustrate the advantages of RPC over traditional APC analysis for studying longitudinal variation in depression screening. We end with concluding remarks in section 5. We consider some extensions of the RPC approach in the appendix (S1 File).

## Methods

### The risk-period-cohort model

The proposed RPC analytic method requires three primary assumptions:

1. Age-related risk is not linearly dependent with chronological age.
2. Chronological age and other risk factors, time periods and birth cohorts necessary to evaluate these relationships are empirically represented in the data.
3. The risk model used to estimate age-related risk is correctly specified. If the risk model is misspecified (e.g. omitted confounding risk factors, risk factors on inappropriate scales, or lack of relevant interaction terms), then we may not be able to accurately estimate age-related risk.

**Fig 1:**
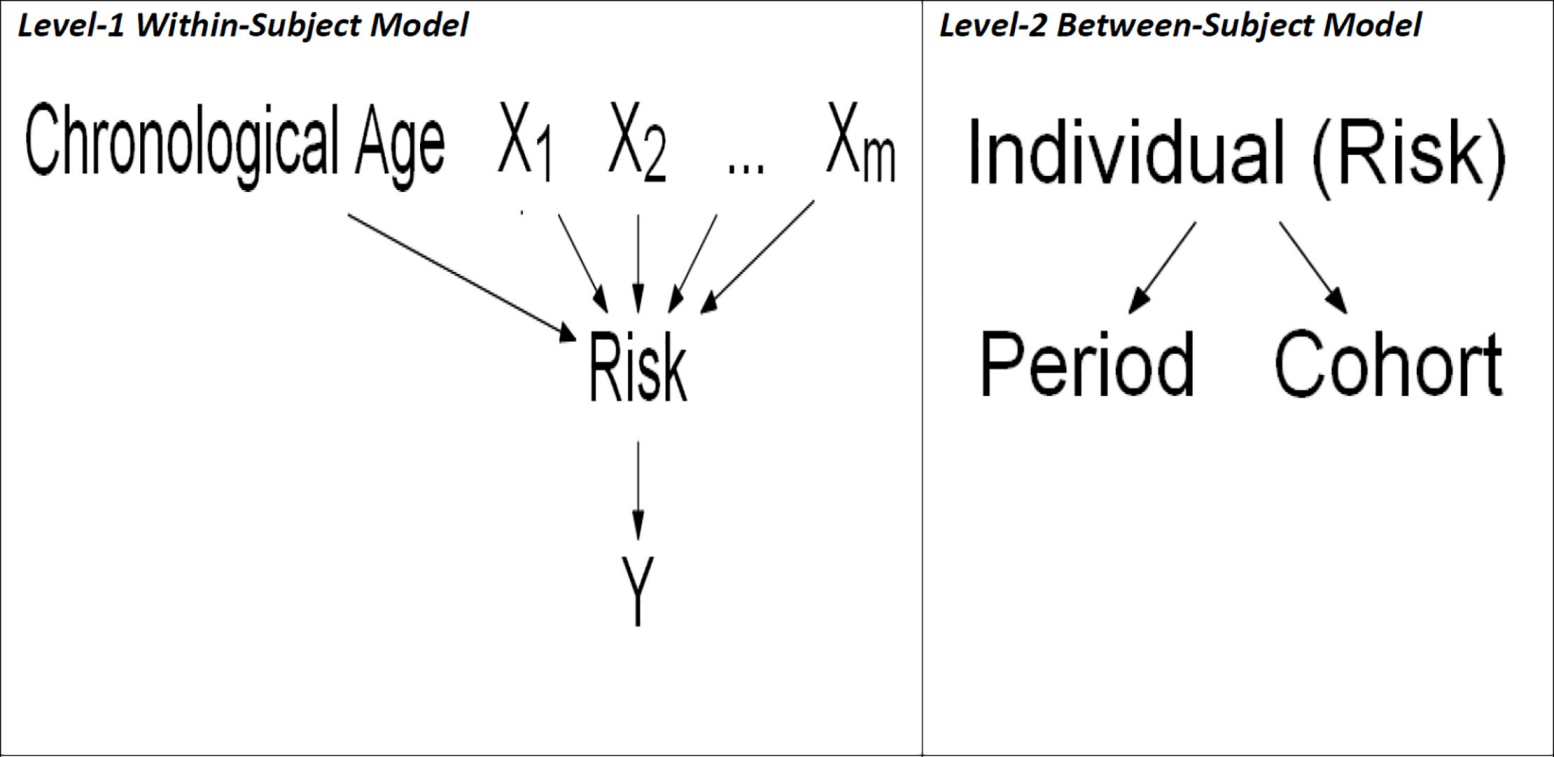
A conceptual diagram for the risk-period-cohort (RPC) model using the hierarchical APC (HAPC) framework. Structural diagram of the HAPC model using RPC analyses (not including a quadratic risk effect on the outcome). This diagram is adapted similarly from work by Bell and Jones (2014)[9]. Each period and cohort will include the spectrum of individuals of different risk levels. However, this is cross-classified since each period does not include all cohort groups and vice versa.

We now describe how RPC can be implemented using repeated measures data across individuals with modifications to extend the Yang and Land’s HAPC framework [4] (see Fig 1).

Let $Y_{i}^{0}$ be a continuous outcome of interest at baseline (hence the superscript) for *i* = 1,…,*n* subjects in our study. Assume chronological age at baseline and **X**_*i*_,a vector of *m* baseline covariates (*X*_1*i*_,*X*_2*i*_,…,*X*_*mi*_)^*T*^, are risk factors for $Y_{i}^{0}$ that describe age-related risk for $Y_{i}^{0}$. An internal risk model with additive effects for chronological age and the set of risk factors **X**_*i*_ is given by the following equation:

$$Y_{i}^{0}=\mu +\beta Chronological\;Age_{i}+\alpha^{T}X_{i}+\varepsilon_{i}$$ (1)

This model describes how variability in the aging process can put a subject at higher or lower risk for the outcome of interest at baseline. Risk in the case of a continuous outcome refers to predicted values of the outcome for each subject based on a fitted risk equation (e.g., via multiple regression or a machine learning algorithm). While Eq (1) can be generalized to other outcome types (e.g., binary, count and time-to-event outcome types, as described in “Extensions of the Risk Modeling Approach” in the Supporting Information, S1 File) and other age-related risk structures (e.g., complex parametric effects involving interactions, nonlinear effects, non-parametric structures, autocorrelated error terms, and/or stratification to allow for heterogeneity across pre-defined groups), in this basic representation we include a residual error term assumed to be independent and identically distributed with zero mean and positive variance. The resulting multidimensional age-related risk index encapsulates predictive information embedded in each subjects’ chronological age at baseline within the context of effects associated with other relevant risk factors:

$$Risk_{i}={\hat{Y}}_{i}^{0}=\hat{\mu }+\hat{\beta }Chronological\;Age_{i}+{\hat{\alpha }}^{T}X_{i}$$ (2)

We can thus obviate the identification problem in traditional APC analyses by substituting the age-related risk dimension defined in (2) in place of chronological age (hence the RPC model), as long as we satisfy our three basic assumptions. *Assumption 1* leads to the necessary condition for RPC that chronological age and age-related risk are not linearly dependent (perfectly collinear) or near linearly dependent (approaching collinearity as the sample size grows infinitely larger). This criterion can be restated formally as an achievement of the requirement that the absolute value of the correlation between chronological age and age-related risk approaches a number strictly less than one as our sample size grows infinitely larger:

$$\underset{n\to \infty }{\lim}\left|\mathrm{cor}\left(Chronological\;Age,Risk\right)\right|\to 1-\delta_{n}$$ (3)

where 0<*δ*_*n*_≤1 as *n*→∞. By having to satisfy *assumption 1* we have eliminated most of the trivial cases (e.g., chronological age uniquely determines risk) where this correlation in (3) is expected to approach one as our sample size grows larger (see simulation study 1).

We can then, alternately as an example, perform longitudinal RPC analyses with the repeated measures outcome of interest by including age-related risk, period and cohort in the proposed Hierarchical APC framework of Yang and Land [4] (see Fig 1):

$$\begin{array}{l} \mathrm{Level}‐1\;\mathrm{within}‐\mathrm{subject}\;\mathrm{model}\\ Y_{ijk}=\alpha_{jk}+\beta_{1jk}Risk_{i}+\varepsilon_{ijk},\\ \mathrm{Level}‐2\;\mathrm{between}‐\mathrm{subject}\;\mathrm{model}\\ \alpha_{jk}=\eta_{0}+\tau_{0j}+\lambda_{0k} \end{array}$$ (4)

for *i* = 1,…,*n*_*jk*_ individuals within the *j*th period for *j* =1,…,*J* time period and the *k*th cohort for *k* = 1,…,*K* birth cohort. There is a Level-1 residual error term *ε*_*ijk*_. The Level-2 model defines the group-level effect *α*_*jk*_ as the sum of the intercept in the Level-1 model by an intercept term *η*_0_ and random effects for each period *τ*_0*j*_ and cohort *λ*_0*k*_. The period, cohort and Level-1 residuals are each assumed to be independent and identically distributed with zero mean and positive variance.

### Stability of results using the RPC method

Above, we described three assumptions of RPC. Satisfying the three assumptions is imperative for obtaining a credible solution (e.g. age-related risk heterogeneity in simulation study 2). Further, if the correlation between chronological age and age-related risk is very high, say 0.97, then serious multicollinearity exists between age-related risk, period and cohort effects that could lead to less reliable results. We suggest that this correlation should be less than or equal to 0.85 [23] to avoid modeling a multicollinear system of age-related risk, period and cohort variables. We show that in practical scenarios similar to our simulation studies, this correlation will most likely be less than or equal to 0.85 in satisfying *assumption 1*. If, on the other hand, risk is relatively independent from chronological age (with a low absolute correlation between risk and chronological age), then the effect of chronological age is small (assuming the risk model has been internally derived). As a result, one might either want to remove age from the model entirely or continue to use risk to describe the aging process, given phenomena related to growing older in the research study is relatively independent of chronological age.

We also importantly note that *assumption 3* for correct model specification should be interpreted in accordance with previous findings on internal risk modeling [19–22]. These findings suggest that if an internal risk model performs well in comparison to existing external models based on measures of predictive accuracy, then there might be clinical utility to the internal risk model. While externally-derived risk models may reasonably be considered if they perform accurately for the sample at hand, internal risk models maximize internal risk performance and for this reason we favor this approach.

In the APC literature in many APC models using repeated measures data across individuals, as mentioned, chronological age at baseline is often treated as a fixed effect while variables representing time period and birth cohort are random effects [4]. As a result in the RPC approach we create an age-related risk index using baseline measures to be used in place of chronological age in a similar manner in such APC models. The proposed risk modeling approach is not specific to age, in that the modeler can employ the same technique to build risk indexes that include period or cohort effects instead of age. Since age, period and cohort effects vary from each other and outcomes of interest in systematic ways, it might be reasonable in certain circumstances to consider risk models that have a different set of covariates in creating each of the three risk indices. These indices can then be used in subsequent longitudinal models in any way as fixed, random or mixed effects.

### Predicted chronological age as age-related risk

Another way to conceptualize age-related risk is as a predicted chronological age after accounting for biological and sociological factors that influence the aging process. Predicted chronological age can be quantified by modeling baseline chronological age as a function of other risk factors (e.g., using multivariable linear regression).

An advantage of using predicted chronological age is that it is calculated on a similar scale as chronological age and as a result is a widely interpretable metric. A disadvantage is that one might not want to conceptualize age-related risk in terms of predicted chronological age independent of an outcome of interest. For example, in a longitudinal study of cardiovascular-related events we might prefer to calculate a five-year age-related risk for a cardiovascular event.

## Results

### Overview of simulation studies of the RPC approach

We present two Monte Carlo (MC) simulation studies using R, version 3.4.2, in the RStudio integrated development environment. In simulation study 1 we illustrate how the correlation between chronological age and age-related risk varies according to the contribution of chronological age in the internal risk model. In simulation study 2, we assume that our risk model is correctly specified (*assumption 3*) and recover the age effect within the context of the age-related risk index. In that simulation, we also evaluate if we can additionally recover underlying cohort and period effects using the RPC approach. We set our sample size n = 10 000 for each MC replication in our simulation studies so that our results were robust and generalizable for large sample behavior. See Table 1 for the details of how we generated random variables and slope parameter patterns for these simulations.

**Table 1:** Conditions, variables and parameters for simulation studies (N = 10 000, 1000 Monte Carlo replications of each slope parameter pattern).

**Simulation Study 1** Risk prediction model for baseline health state $Y_{i}^{0}$: $Y_{i}^{0}=\mu +\beta Chronological\;Age_{i}+\alpha_{1}SEP_{i}+\alpha_{2}Activity_{i}+\varepsilon_{i}$, *i* = 1,…,10000	
**Variable/Parameter**	**Condition for Simulation**
Intercept (*μ*)	set to 0 without loss of generality
Chronological Age	truncated normal distribution with mean 55, standard deviation 25, lower bounds 30, upper bounds 80; rounded to the nearest integer
SEP	standard normal distribution
Activity	poisson distribution with parameter lambda set to one
Residual error term (*ε*_*i*_)	standard normal distribution
*β*, *α*_1_ and *α*_2_	Each drawn with replacement from the vector (0,0.2,0.4,0.6,0.8,1)^*T*^ for 6^3^ = 216 possible parameter patterns
**Additional Information for Simulation Study 2** APC model for current health state *Y*_*i*_ (under assumption of no period effect): *Y*_*i*_ = *μ*+*βChronological Age*_*i*_+*α*_1_*SEP*_*i*_+*α*_2_*Activity*_*i*_+Γ*Cohort*_*i*_+*ε*_*i*_, *i* = 1,…,10000	
**Variable/Parameter**	**Condition for Simulation**
Period	truncated normal distribution with mean 1980 standard deviation 30, lower bounds 1950, upper bounds 2010
Cohort	Due to linear dependency we calculated for each individual *i* a birth cohort, *Cohort*_*i*_ + *Period*_*i*_−*Chronological Age*_*i*_.
Γ	Additionally drawn with replacement from the vector (0,0.2,0.4,0.6,0.8,1)^*T*^ for 6^4^ = 1296 possible parameter patterns

SEP = Socioeconomic Position; Activity = Physical Activity. We standardized all random variables (Chronological Age, SEP, Activity and Cohort) for simulating the outcomes for baseline and current health state, so these variables were all on a similar scale with the simulated slopes. The coefficients are based on a standardized scale and generally the effects represent null (0.0), small (0.2), moderate (0.4 and 0.6), large (0.8) and very large (1.0) effects.

### Simulation study 1

We simulated a simplified risk prediction model for baseline health state with chronological age and two additional risk factors, socioeconomic position (SEP) and physical activity level and a residual error term assumed to follow a standard normal distribution:

$$Y_{i}^{0}=\mu +\beta Chronological\;Age_{i}+\alpha_{1}SEP_{i}+\alpha_{2}Activity_{i}+\varepsilon_{i}$$ (5)

In this simulation, the aging process in relation to subject-level variation in baseline health state can be described as a function of chronological age, SEP and physical activity level. The distribution of the variables considered in this model as shown in Table 1 was simulated for what we considered practical purposes. We consider a study population with numerical chronological ages from 30 to 80, a normally distributed SEP variable and a variable activity that is non-normally distributed across the study population to consider a measure with skewness in a real population. We can replace these variables without loss of generality since we will be interested in the magnitude of the slopes in (5) (*β*,*α*_1_,*α*_2_) for these covariates, which in this setting is controlled via the simulation conditions independent of the type of variable.

We define an age-related risk index for the fitted value for the outcome, where

$$Risk_{i}=\hat{Y_{{}_{i}}^{0}}=\hat{\mu }+\hat{\beta }Chronological\;Age_{i}+\hat{\alpha_{1}}SEP_{i}+\hat{\alpha_{2}}Activity_{i}$$ (6)

We varied the slopes in (5) (*β*,*α*_1_,*α*_2_) in simulating data to understand how the contribution of the age effect can affect the correlation between chronological age and age-related risk. Loosely, the magnitude of the chosen effects represent null (0.0), small (0.2), moderate (0.4 and 0.6), large (0.8) and very large (1.0) effects on a standardized coefficient scale.

We fit the corresponding risk prediction model for (5) using linear regression. Then, we calculated the age-related risk index using (6). We calculated the size of the chronological age effect as the proportionate chronological age effect in the risk prediction model using the estimated parameters in (6):

$$\mathrm{Proportionate}\;\mathrm{Chronological}\;Age\;Effect=\frac{\hat{\beta }}{\hat{\beta }+\hat{\alpha_{1}}+\hat{\alpha_{2}}}$$ (7)

**Fig 2:**
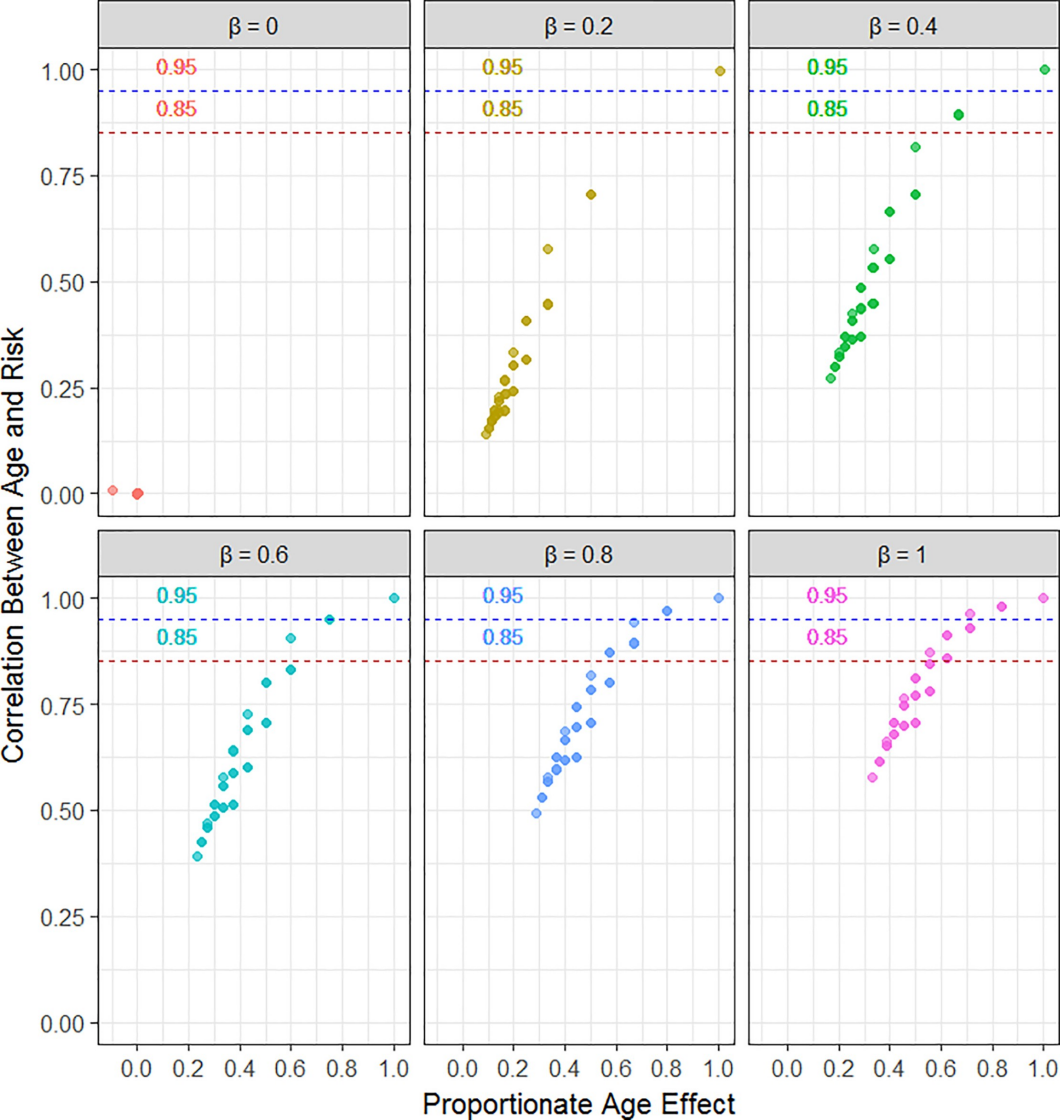
Scatterplots of the correlation between age and risk vs. proportionate chronological age effect grouped by simulated beta value (0,0.2,0.4,0.6,0.8,1). Average results over 1000 Monte Carlo (MC) replications of each of the 216 slope parameter patterns in which N = 10 000 for each MC replication. Results were robust over the MC replications; the empirical standard errors were close to zero for the average correlation of age and risk across all slope parameter patterns and for proportionate age effect with the exception of the trivial case where *β* = 0,*α*_1_ = 0,*α*_2_ = 0 (empirical standard error = 0.06). Proportionate age effect is defined using the formula (1.7). The coefficients are based on a standardized scale and generally the effects represent null (0.0), small (0.2), moderate (0.4 and 0.6), large (0.8) and the largest possible (1.0) effects.

The overwhelming majority of the slope parameter patterns yielded useful age-related risk indices, containing information regarding the extent to which chronological age determines age-related risk (within the context of other effects; see Fig 2). The simulation showed that the correlation between chronological age and risk generally goes down or up according to contribution of chronological age in the risk prediction model. Table 2 includes all cases where the correlation between chronological age and age-risk were above the 0.85 threshold (29 cases). We found that the correlation between chronological age and risk was above the 0.85 threshold whenever the proportionate chronological age effect was greater than 0.60 (24/29 cases); the proportionate chronological age effect was still quite large, between 0.55 and 0.59, for the other five cases (see Table 2).

**Table 2:** Simulated slope parameter patterns in which the average correlation between chronological age and age-related risk was greater than or equal to 0.85.

*Chronological age* (*β*)	*SEP*(*α*_1_)	*ACTIVITY*(*α*_2_)	*CORRELATION BETWEEN CHRONOLOGICAL AGE AND AGE-RELATED RISK*	*PROPORTIONATE CHRONOLOGICAL AGE EFFECT*
***0*.*2***	***0***	***0***	***1***	***1*.*01***
***0*.*4***	***0***	***0***	***1***	***1***
***0*.*6***	***0***	***0***	***1***	***1***
***0*.*8***	***0***	***0***	***1***	***1***
***1***	***0***	***0***	***1***	***1***
***1***	***0***	***0*.*2***	***0*.*98***	***0*.*83***
***1***	***0*.*2***	***0***	***0*.*98***	***0*.*83***
***0*.*8***	***0***	***0*.*2***	***0*.*97***	***0*.*8***
***0*.*8***	***0*.*2***	***0***	***0*.*97***	***0*.*8***
***1***	***0*.*2***	***0*.*2***	***0*.*96***	***0*.*71***
***0*.*6***	***0***	***0*.*2***	***0*.*95***	***0*.*75***
***0*.*6***	***0*.*2***	***0***	***0*.*95***	***0*.*75***
***0*.*8***	***0*.*2***	***0*.*2***	***0*.*94***	***0*.*67***
***1***	***0***	***0*.*4***	***0*.*93***	***0*.*71***
***1***	***0*.*4***	***0***	***0*.*93***	***0*.*71***
***1***	***0*.*2***	***0*.*4***	***0*.*91***	***0*.*62***
***1***	***0*.*4***	***0*.*2***	***0*.*91***	***0*.*62***
***0*.*6***	***0*.*2***	***0*.*2***	***0*.*9***	***0*.*6***
***0*.*4***	***0***	***0*.*2***	***0*.*89***	***0*.*67***
***0*.*4***	***0*.*2***	***0***	***0*.*89***	***0*.*67***
***0*.*8***	***0***	***0*.*4***	***0*.*89***	***0*.*67***
***0*.*8***	***0*.*4***	***0***	***0*.*89***	***0*.*67***
***0*.*8***	***0*.*2***	***0*.*4***	***0*.*87***	***0*.*57***
***0*.*8***	***0*.*4***	***0*.*2***	***0*.*87***	***0*.*57***
***1***	***0*.*4***	***0*.*4***	***0*.*87***	***0*.*56***
***1***	***0***	***0*.*6***	***0*.*86***	***0*.*62***
***1***	***0*.*6***	***0***	***0*.*86***	***0*.*63***
***1***	***0*.*2***	***0*.*6***	***0*.*85***	***0*.*56***
***1***	***0*.*6***	***0*.*2***	***0*.*85***	***0*.*56***

Proportionate chronological age effect is defined using the formula (7). The coefficients are based on a standardized scale and generally the effects represent null (0.0), small (0.2), moderate (0.4 and 0.6), large (0.8) and the largest possible (1.0) effects.

When chronological age correlated with the outcome only weakly, then the age-related risk index was close to linear independence from chronological age (see Fig 2 when *β* = 0). However, the sole outlying point in Fig 2 for *β* = 0 (proportionate chronological age effect is incongruent with the near zero correlation between chronological age and age-related risk due to noise) is the trivial case where all slope parameters are zero, violating *assumption 1*.

### Simulation study 2

For simplicity, we assumed that current health state can be simulated in the same manner as baseline health state from (5) with the exception that current health state also considers a cohort variable:

$$Y_{i}=\mu +\beta Chronological\;Age_{i}+\alpha_{1}SEP_{i}+\alpha_{2}Activity_{i}+\Gamma Cohort_{i}+\varepsilon_{i}$$ (8)

We fit the model for (8) using linear regression. We assumed no period effect in this model in order to study the RPC model’s capacity for recovering null effects (in addition to values of the parameter Γ). We also used the simulated data based on each slope parameter pattern to fit respective RPC models, incorporating the age-related risk index from Eq (6):

$$Y_{i}=\mu^{*}+\beta^{*}Risk_{i}+\Gamma^{*}Cohort_{i}+\Lambda^{*}Period_{i}+\varepsilon_{i}$$ (9)

Assuming we have recovered the age effect within the age-related risk index, if we could satisfactorily recover the cohort and period effects using RPC, we would expect Γ−Γ* = 0 and Λ* = 0. We flagged cases where the average absolute value of either of these two estimated values over the MC replications of each slope parameter pattern was greater than a trivially small effect size (0.01). In these cases, we might fail to precisely recover the simulated cohort effect and falsely identify some possibility of a nonexistent period effect.

We found that the RPC approach estimates accurately both the simulated cohort and period effects using our criteria for 1266 of the 1296 (98%) slope parameter patterns we tested. The 30 cases where period and cohort effects were not precisely estimated in the RPC model (see Table 3) consisted of all the trivial cases where chronological age was the only significant risk factor in the internal risk model (*β*>0,*α*_1_ = 0,*α*_2_=0). The bias grew with the size of the chronological age parameter *β*. Therefore, in imposing the assumption of age-related risk heterogeneity (*assumption 1*) we would be able to recover all additional cohort and period effects using RPC in our simulated nontrivial test cases.

**Table 3:** Problematic slope parameter patterns in simulation study 2.

Chronological Age *β*	SEP *α*_1_	Activity *α*_2_	Cohort Γ	Cohort Effect Difference	RPC Period Effect
0.2	0	0	0	0.29	0.23
0.2	0	0	0.2	0.31	0.24
0.2	0	0	0.4	0.30	0.23
0.2	0	0	0.6	0.20	0.15
0.2	0	0	0.8	0.32	0.25
0.2	0	0	1	0.30	0.23
0.4	0	0	0	0.51	0.40
0.4	0	0	0.2	0.63	0.48
0.4	0	0	0.4	0.61	0.47
0.4	0	0	0.6	0.66	0.51
0.4	0	0	0.8	0.66	0.51
0.4	0	0	1	0.61	0.46
0.6	0	0	0	0.96	0.74
0.6	0	0	0.2	0.89	0.68
0.6	0	0	0.4	0.92	0.71
0.6	0	0	0.6	0.99	0.76
0.6	0	0	0.8	0.93	0.71
0.6	0	0	1	0.92	0.70
0.8	0	0	0	1.35	1.03
0.8	0	0	0.2	1.25	0.96
0.8	0	0	0.4	1.27	0.97
0.8	0	0	0.6	1.16	0.89
0.8	0	0	0.8	1.34	1.03
0.8	0	0	1	1.17	0.90
1	0	0	0	1.71	1.32
1	0	0	0.2	1.88	1.45
1	0	0	0.4	1.65	1.27
1	0	0	0.6	1.51	1.16
1	0	0	0.8	1.52	1.16
1	0	0	1	1.40	1.08

### Real-world application of the RPC approach to model changes in depression screening outcomes from 2005–2016

We performed both traditional APC and RPC analysis on an illustrative example to demonstrate the potential consequences of not being able to estimate all levels of age, period and cohort effects in real settings.

The National Health and Nutrition Examination Survey (NHANES) is a program of the National Center for Health Statistics that began in 1960. The objective of the NHANES is to assess the health and nutritional status of individuals in the United States. The NHANES is a cross-sectional collection of surveys and other health examination data for a nationally representative sample of the resident, civilian, non-institutionalized U.S. population with approximately 5,000 individuals sampled each year [24, 25].

The inclusion criteria for our illustrative example was having a recorded Patient Health Questionnaire (PHQ)-9 total score [26] from one of six time periods (2005–2006, 2007–2008, 2009–2010, 2011–2012, 2013–2014, 2015–2016) Among the 31191 individuals meeting these criteria, the median chronological age was 47, 51.5% were female, 46.9% were non-Hispanic White, and 64.2% had no comorbidities. The median PHQ-9 total score was 2, and 8.7% of individuals screened positive for depressive symptoms (PHQ-9 total score ≥ 10).

We created an age-related risk index using predicted chronological age. The internal risk model for chronological age included sex, number of comorbidities (ranging from 0 to 11), race/ethnicity (Hispanic, Non-Hispanic White, Non-Hispanic Black and Other Race), ratio of family income to poverty level, and social support (year = married or living with partner, no = widowed, divorced, separated or never married) as predictor variables. We performed listwise deletion on missing values on the covariates for 27496 complete cases. In our risk model, all variables were statistically significant (p < 0.05) and the model R-squared and adjusted R-squared were both 0.23. The correlation between age-related risk and chronological age was 0.48, thus fitting well within our conditions for this correlation as described in simulation study 1.

In a traditional APC approach, we regressed the PHQ-9 total score on chronological age, time period and birth cohort. In the RPC approach we regressed PHQ-9 total score on age-related risk, time period and birth cohort. For approximating a birth cohort for each individual, we used the even year in each two-year range (2006, 2008, 2010, 2012, 2014, 2016) as the time period. We categorized both time period and birth cohort. We then grouped together birth cohorts every five years for our regression modeling. Due to the skewness of the PHQ-9 in our study population, we used a logarithmic + 1 transformation on this outcome.

The APC approach was only able to estimate time period and birth cohort because we categorized birth cohort by five-year ranges and removed levels that would make it linearly dependent with chronological age and time period; nonetheless there was evidence of variance inflation (i.e., larger standard errors) for period and cohort effects within the APC model relative to those estimated in the RPC model (see Table 4). The RPC model additionally resulted in improved model fit statistics (R^2^ and adjusted R^2^) relative to the APC model.

**Table 4:** Application of the RPC approach and a traditional APC approach on NHANES survey data to evaluate time-varying trends in depression screening from 2005–2016 (N = 27496).

	RPC			Traditional APC		
	R^2^ = 0.046; Adjusted R^2^ = 0.046			R^2^ = 0.008; Adjusted R^2^ = 0.007		
	Estimate	SE	p	Estimate	SE	p
(Intercept)	-0.426	0.070	<0.001	0.614	0.326	0.060
***Chronological Age***	—	—	—	0.003	0.004	0.460
***Age-related Risk***	0.023	0.001	<0.001	—	—	—
***Period*** (Reference 2005–2006)						
2007–2008	0.109	0.018	<0.001	0.113	0.020	<0.001
2009–2010	0.095	0.018	<0.001	0.099	0.024	<0.001
2011–2012	0.029	0.019	0.125	0.026	0.030	0.386
2013–2014	0.053	0.019	0.004	0.064	0.035	0.070
2015–2016	0.053	0.019	0.006	0.068	0.043	0.108
***Cohort*** (Reference 1921)						
1926–1930	-0.111	0.068	0.101	-0.106	0.073	0.147
1931–1935	0.018	0.065	0.776	0.044	0.078	0.567
1936–1940	0.025	0.064	0.695	0.031	0.087	0.721
1941–1945	0.056	0.064	0.382	0.050	0.102	0.624
1946–1950	0.166	0.063	0.009	0.149	0.117	0.201
1951–1955	0.280	0.063	<0.001	0.241	0.133	0.070
1956–1960	0.289	0.063	<0.001	0.239	0.150	0.110
1961–1965	0.341	0.063	<0.001	0.262	0.167	0.118
1966–1970	0.328	0.063	<0.001	0.236	0.186	0.203
1971–1975	0.315	0.063	<0.001	0.221	0.203	0.276
1976–1980	0.374	0.063	<0.001	0.271	0.222	0.222
1981–1985	0.367	0.063	<0.001	0.255	0.240	0.288
1986–1990	0.457	0.063	<0.001	0.334	0.258	0.196
1991–1996	0.500	0.068	<0.001	0.372	0.277	0.180

Abbreviations: RPC = risk-period-cohort; APC = age-period-cohort; SE = standard error; p = p-value. In an alternative specification, we categorized every level of birth cohort and thus had to leave out a level in order to resolve the identifiability problem. The substantive results and conclusions for this alternative specification regarding age, period and cohort effects were similar however as the traditional APC approach presented in Table 4.

Age-related risk was significantly associated with depression screening in the RPC model, while chronological age was not significantly associated with depression screening in the traditional APC model. Point estimates for period effects in the two models were relatively similar, although the RPC model showed a significantly positive association between time periods for 2013–2014 and 2015–2016 (reference level 2005–2006) with PHQ-9 total score while the traditional APC model did not. The birth cohort point estimates were in a similar direction between the two models. However, the traditional APC model had lower point estimates (for the most part) and larger standard errors and did not have a significant birth cohort effect. Of note in the RPC model, there was a strong birth cohort trend beginning in 1946–1950 associated with an increase in depression scores. This positive association grows in magnitude across younger birth cohorts.

**Fig 3:**
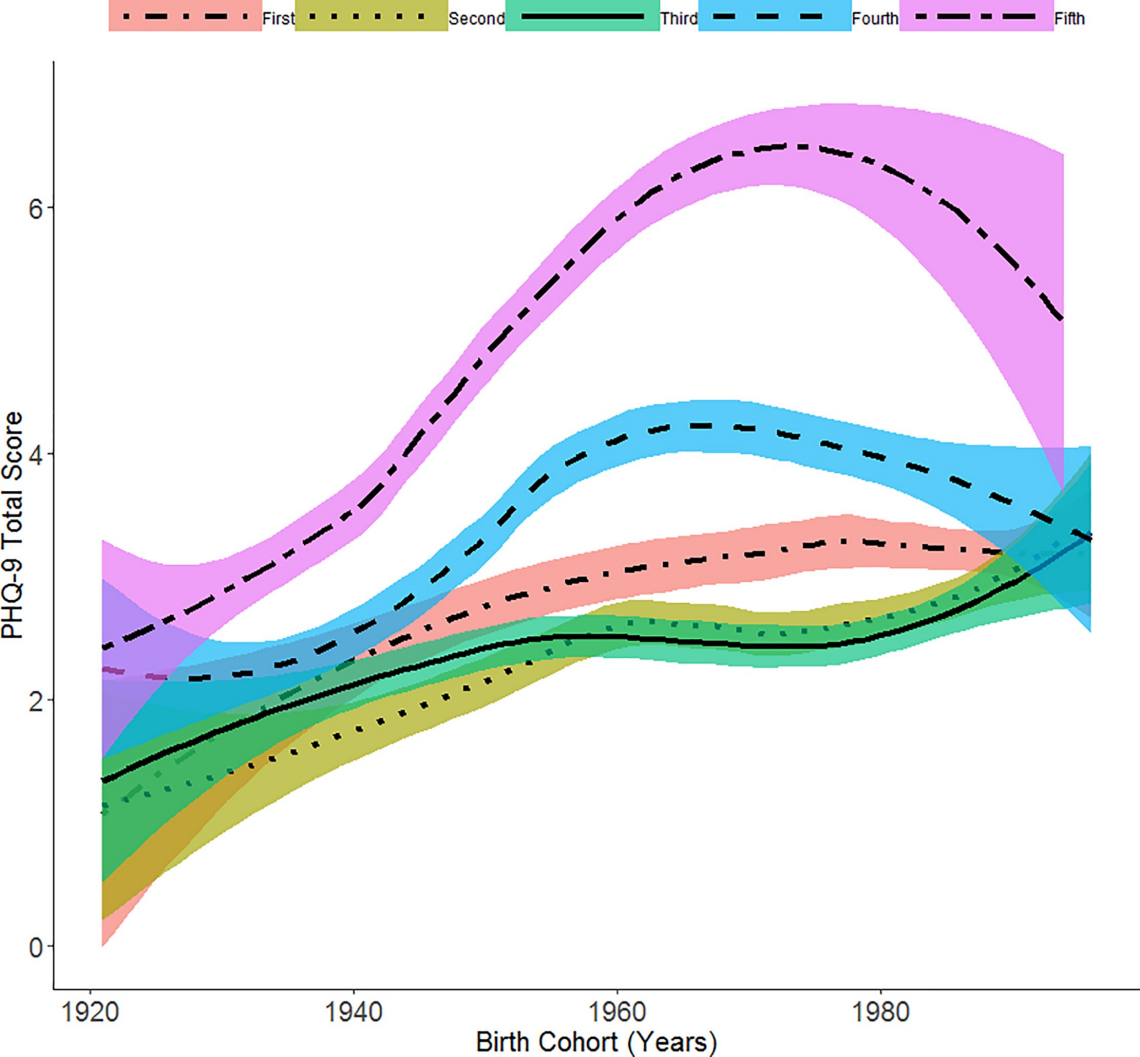
Relationship between PHQ-9 total score and birth cohort by quintiles of age-related risk using local regression smoothing (N = 27496). Shaded region in each plot represents a 95% Confidence Interval.

In Fig 3 we graphically depict the relationship between PHQ-9 total score and birth cohort grouped by quintiles of age-related risk. The figures shows, with considerable variability across age-related risk groups, an increase in the average PHQ-9 total score in moving across the x axis to younger birth cohorts. The increasing slope is most prominent for individuals in the fifth quintile (highest age-related risk) before data becomes more sparse in later birth cohorts.

We re-ran the APC model including the explanatory variables used in the risk model as covariates. The model fit improved substantially (R^2^ and adjusted R^2^ both equal to 0.12). However, the standard errors in general were still larger than in the RPC model. In this model one still needs to force a constraint in order to estimate the age, period and cohort effects due to linear dependency. The major difference in the results from this APC analysis (incorporating covariates) in comparison to the traditional APC model reported in Table 4 was that a significant (p-value ranging from 0.01 to 0.05) birth cohort effect was detected between 1951 and 1970. This effect was then no longer significant for the post-1970 birth cohorts.

For further confirmation by comparison to a mixed effects approach, we re-ran both the RPC and APC model using a multilevel framework. In the RPC multilevel model we included a fixed effect for risk and in the APC multilevel model we included fixed effects for chronological age and quadratic chronological age. Both models included random effects for period and cohort. The mixed effects results paralleled the results for the fixed effects regression models: the RPC model detected a significant positive age-related risk effect and positive cohort effect from 1951 onward, while the APC model did not detect an age or cohort effect.

### Future work for evaluating RPC effects in a longitudinal study of cardiovascular-related events

Our real data application with the NHANES survey data used different waves of cross-sectional data. Thus, we were not able to evaluate age, period and period effects on the same individuals across time. In a future study we will evaluate age-related risk, period and cohort effects for cardiovascular-related events using repeated measures data across individuals. A possible hypothesis would be that meaningful RPC effects would be observed because older birth cohorts were at higher risk for cardiovascular outcomes, including mortality, in the 1980s compared to today due to higher prevalence of smoking and the advent of a variety of effective clinical and medical interventions since the 1980s (e.g. statins) [27].

## Conclusions

Age-period-cohort (APC) models have been proposed as a longitudinal modeling solution that can decompose data trends for time-varying events into age, period and cohort effects. However, due to the identification problem, researchers have been hamstrung by challenges in recovering underlying APC effects arising from the inherent collinearity of these factors. Prior longitudinal studies suffer from a potentially critical limitation of understanding the full range of longitudinal variation when not properly examining all age, period and cohort effects [9, 11–15, 27–29]. Our risk-period-cohort (RPC) method provides a straightforward way to understand such longitudinal variation by obviating the identification problem.

Our RPC approach essentially transforms chronological age into a multidimensional hybrid measure to describe age-related risk. Further, the RPC approach allows for contextualizing APC effects in relation to clinical outcomes and other risk factors while addressing different sources of subject heterogeneity. As such, we believe that the RPC framework can be usefully applied for many epidemiological studies of human development and gerontology involving large observational data sources, in that it allows for examining sources of variation over broad time durations.

Our first simulation study revealed that in most of our tested cases, the age-related risk index provided for reasonable levels of multicollinearity among risk, period and cohort effects. The risk index also informs the researcher about the size of the chronological age effect in contributing to age-related risk. In using the RPC approach, as revealed in our simulation study 1, it is important to check (1) the correlation between the age-related risk index and chronological age and (2) the size of the chronological age effect (i.e. proportionate chronological age effect) in the risk prediction model. In practice, model adjustments in line with theory and prior empirical evidence might be considered to optimize these two indices before performing subsequent longitudinal RPC analyses. Although we anticipate that the values of these two indices should generally be below acceptable thresholds described above, investigators might employ remedial measures when the indices are above those thresholds (e.g., inclusion of additional clinically relevant risk factors for age-related risk in the risk prediction model when such factors are available).

Existing methods that attempt to resolve the identification problem may fail to recover underlying APC effects due to variance inflation resulting from multicollinearity of these factors. Our second simulation study showed in our test cases, given that the internal risk model is correctly specified and recovers the age effect within the age-related risk index, that the RPC approach was able to additionally recover both the simulated cohort and period effects in all nontrivial cases. These simulation studies showed the necessity of the *assumption 1* of age-related risk heterogeneity. The simulation results showed that the RPC model should be useful for many applied problems. However, there are other scenarios encountered in practice not covered in our simulation studies that will need to be studied further.

Our real data application of the RPC approach on the NHANES survey data illustrated the advantages of being able to recover all levels of APC effects without making constraints to resolve the identifiability problem. Smaller standard errors were observed in the RPC model as compared to a traditional APC model under different specifications. Of primary concern was that the traditional APC model may have missed substantively important longitudinal variation in depression screening between 2005–2016. The RPC model found a significant age effect and a cohort effect beginning around 1946–1950 and continuing across subsequent birth cohorts that was absent from the traditional APC models.

In this paper we have described the RPC approach for obviating identifiability in APC models with broad implications for accounting for time-varying events that impact longitudinal studies. In future studies we will seek to apply this approach using longitudinal data from national cohorts and from electronic health records. Such future work is needed to examine the empirical value of the RPC approach using repeated measures data on individuals.

## Supporting information

S1 File Appendix. (PDF)

S2 File RPC R code for the two simulation studies and NHANES survey data application. (ZIP)
