# Supplementary material for: Risk-period-cohort approach for averting identification problems in longitudinal models
Source: PLoS One. 2019 Jul 10;14(7):e0219399. doi: 10.1371/journal.pone.0219399 (PMC6620014; doi:10.1371/journal.pone.0219399)
Supplement: S1 File — (PDF) [file pone.0219399.s001.pdf]

Appendix

Extensions of the risk modeling approach

Non-linear outcomes

We can propose the following internal risk model for different types of outcome (i.e. binary, continuous, count) as an extension of the continuous outcome case using an appropriate link function $f\left(Y_i^0 \middle| Chronological\ Age_i, \mathbf{X}_i\right)$:

$$E\left[\ f\left(Y_i^0 \middle| Chronological\ Age_i, \mathbf{X}_i\right)\right] = \mu + \beta Chronological\ Age_i + \boldsymbol{\alpha}^{\mathbf{T}}\mathbf{X}_i \tag{1}$$

The resulting age-related risk index for each individual i can be calculated using the fitted values:

$$Risk_i = f\left(Y_i^0 \middle| Chronological\ Age_i, \mathbf{X}_i\right) = \mu + \beta Chronological\ Age_i + \boldsymbol{\alpha}^{\mathbf{T}}\mathbf{X}_i \tag{2}$$

This strategy can be applied for different outcomes (i.e. time-to-event, nonparametric) outside of the generalized linear model framework. For example, for time-to-event outcomes while using Cox's model, an age-related risk index can be derived in terms of individual risk of the event compared to individual baseline risk:

$$Risk_i = \exp\left(\beta Chronological\ Age_i + \boldsymbol{\alpha}^{\mathbf{T}}\mathbf{X}_i\right) \quad (3)$$

This formula (3) does not involve the baseline hazard function.

Defining the time to event outcome in the risk model differently than the RPC model is a necessary exercise. One way to do this would be to use a counting process approach [1]. Assume we have a time to event outcome that we have observed in an interval from baseline up to time *t*. We could define the outcome from baseline up to time *s* in the internal risk prediction model, where $0 \le s < t$. Then, independently, we could evaluate the outcome from time *s* to time *t* in the RPC model. This approach could be implemented when time *s* was over a short time frame where we might observe age effects but not cohort and period effects (e.g. estimating 5 year risk for cardiovascular-related events [2]), while time *s* to time *t* was over a longer time frame (e.g. 15 years) in which we might observe period and cohort differences.

Latent variable modeling

A latent variable modeling approach [3-5] could be used to describe age-related risk independent of the outcome of interest [6]. For example, the underlying latent trait of risk for heart disease is influenced by levels of the risk factors (formative indicators) that collectively describe the aging process in the path diagram in S1 Fig in a given population and data set. A principal components analysis approach could be used for constructing an individualized risk index by using the first principal component.

S1 Fig. Path Diagram for one factor formative structure of risk of developing heart disease.

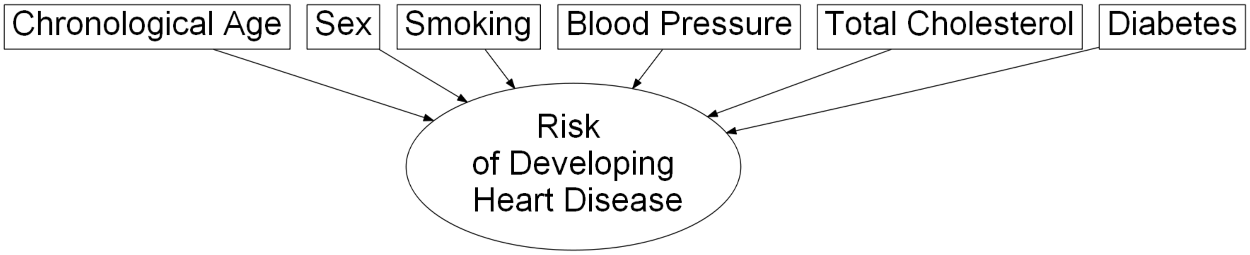

The measurement model alone with formative indicators corresponding to S1 Fig is not identifiable using a structural equation modeling framework. However, the measurement model approach to estimating risk does lend itself to simultaneous estimation of the underlying latent trait of risk and period and cohort within a multilevel structural equation modeling framework [3-5]. For example, corresponding to this example in S1 Fig, the structural model could evaluate the influence of risk of developing heart disease (a latent variable) on cardiovascular-related events in level-1 while including level-2 random effects for period and cohort.

Other latent variable approaches, using finite mixture modeling techniques, can be used for evaluating heterogeneity in the data structure [7-11] . Finite mixture modeling techniques use statistical modeling for identifying unobserved subpopulations within a study population. Thus, for example, using such methodology (e.g. latent profile analysis) individuals could be classified into different age-related risk groups based on a set of risk factors including chronological age independent of an outcome of interest. In a latent growth model using such methodology, individuals could also be classified into subgroups based on trajectory changes for an outcome of interest (i.e. growth mixture

modeling). One could then determine the influence of risk, period and cohort variables on subgroup membership.

References

1. Kalbfleisch JD, Prentice RL. The statistical analysis of failure time data: John Wiley & Sons; 2011.

2. Dalton JE, Perzynski AT, Zidar DA, et al. Accuracy of cardiovascular risk prediction varies by neighborhood socioeconomic position: A retrospective cohort study. Annals of Internal Medicine. 2017;167(7):456-64. doi: 10.7326/M16-2543.

3. Bollen K. Structural equations with latent variables. New York, NY: Wiley; 1989.

4. Kline RB. Principles and practice of structural equation modeling: Guilford press; 2011.

5. Brown TA. Confirmatory factor analysis for applied research: Guilford Publications; 2014.

6. Gunzler DD, Morris N. A tutorial on structural equation modeling for analysis of overlapping symptoms in co-occurring conditions using MPlus. Statistics in Medicine. 2015;34(24):3246-80.

7. Zou Y, Ash JE, Park B-J, Lord D, Wu L. Empirical Bayes estimates of finite mixture of negative binomial regression models and its application to highway safety. Journal of Applied Statistics. 2018;45(9):1652-69. doi: 10.1080/02664763.2017.1389863.

8. Kappe E, DeSarbo WS, Medeiros MC. A Smooth Transition Finite Mixture Model for Accommodating Unobserved Heterogeneity. Journal of Business & Economic Statistics. 2019:1-24.

9. Gunzler DD, Morris N, Perzynski A, Ontaneda D, Briggs F, Miller D, et al. Heterogeneous depression trajectories in multiple sclerosis patients. Multiple Sclerosis and Related Disorders. 2016;9:163-9.

10. Lanza ST, Rhoades BL. Latent class analysis: An alternative perspective on subgroup analysis in prevention and treatment. Prevention Science. 2013;14(2):157-68.

11. Gunzler D, Sajatovic M, McCormick R, Perzynski A, Thomas C, Kanuch S, et al. Psychosocial Features of Clinically Relevant Patient Subgroups With Serious Mental Illness and Comorbid Diabetes. Psychiatric Services. 2016; 68(1):96-9.
