# Supplementary material for: Risk-period-cohort approach for averting identification problems in longitudinal models
Source: PLoS One. 2019 Jul 10;14(7):e0219399. doi: 10.1371/journal.pone.0219399 (PMC6620014; doi:10.1371/journal.pone.0219399)

RPC Real Data Application NHANES

June 11, 2019

## NHANES Survey Data

R program for Real Data Application with NHANES survey data

library("openxlsx")

## Warning: package 'openxlsx' was built under R version 3.5.3

NHANES=read.xlsx('H://NHANES PHQ9.xlsx', sheet = 1)

#distribution of the PHQ-9 in NHANES from 2005-2016
summary(NHANES$PHQ9.Total)

## Min. 1st Qu. Median Mean 3rd Qu. Max. NA's
## 0.000 0.000 2.000 3.193 4.000 27.000 3772

hist(log(NHANES$PHQ9.Total))

NHANES1 <- as.data.frame(unclass(NHANES))

NHANES1$Race=factor(NHANES1$Race.Ethnicity, labels=c("Hispanic","Non-Hispanic White","Non-Hispanic Black","Other Race"))

NHANES1$Race1=factor(NHANES1$Race, levels=c("Non-Hispanic White","Hispanic","Non-Hispanic Black","Other Race"))

NHANES1$Gender <- factor(NHANES1$Gender, labels=c("Male", "Female"))

#listwise delete all missing data
NHANES1=na.omit(NHANES1)

#age-related risk model using chronological age as the outcome
risk=lm(Age~Gender+Race1+Family.PIR+X..of.comorbidities+Social.Support, data=NHANES1)
summary(risk)

##
## Call:
## lm(formula = Age ~ Gender + Race1 + Family.PIR + X..of.comorbidities +
## Social.Support, data = NHANES1)
##
## Residuals:
## Min 1Q Median 3Q Max
## -74.307 -12.881 -1.004 11.747 45.968
##
## Coefficients:
## Estimate Std. Error t value Pr(>|t|)
## (Intercept) 41.02840 0.28172 145.634 < 2e-16 ***
## GenderFemale -0.70762 0.19219 -3.682 0.000232 ***
## Race1Hispanic -2.21393 0.24774 -8.937 < 2e-16 ***
## Race1Non-Hispanic Black -1.90624 0.25532 -7.466 8.5e-14 ***
## Race1Other Race -3.85698 0.34960 -11.033 < 2e-16 ***
## Family.PIR 0.90748 0.06190 14.661 < 2e-16 ***
## X..of.comorbidities 7.18236 0.08296 86.574 < 2e-16 ***
## Social.Supportyes 2.13598 0.20197 10.576 < 2e-16 ***
## ---
## Signif. codes: 0 '***' 0.001 '**' 0.01 '*' 0.05 '.' 0.1 ' ' 1
##
## Residual standard error: 15.83 on 27488 degrees of freedom
## Multiple R-squared: 0.2344, Adjusted R-squared: 0.2342
## F-statistic: 1202 on 7 and 27488 DF, p-value: < 2.2e-16

#create age-related risk index using predicted chronological age
NHANES1$RI=fitted(risk)

#create a categorical variable grouping cohort by five years
NHANES1$cohort.range[NHANES1$cohort>=1921 & NHANES1$cohort<=1925]="1921-1925"
NHANES1$cohort.range[NHANES1$cohort>=1926 & NHANES1$cohort<=1930]="1926-1930"
NHANES1$cohort.range[NHANES1$cohort>=1931 & NHANES1$cohort<=1935]="1931-1935"
NHANES1$cohort.range[NHANES1$cohort>=1936 & NHANES1$cohort<=1940]="1936-1940"
NHANES1$cohort.range[NHANES1$cohort>=1941 & NHANES1$cohort<=1945]="1941-1945"

NHANES1$cohort.range[NHANES1$cohort>=1946 & NHANES1$cohort<=1950]="1946-1950"
NHANES1$cohort.range[NHANES1$cohort>=1951 & NHANES1$cohort<=1955]="1951-1955"
NHANES1$cohort.range[NHANES1$cohort>=1956 & NHANES1$cohort<=1960]="1956-1960"
NHANES1$cohort.range[NHANES1$cohort>=1961 & NHANES1$cohort<=1965]="1961-1965"

NHANES1$cohort.range[NHANES1$cohort>=1966 & NHANES1$cohort<=1970]="1966-1970"
NHANES1$cohort.range[NHANES1$cohort>=1971 & NHANES1$cohort<=1975]="1971-1975"
NHANES1$cohort.range[NHANES1$cohort>=1976 & NHANES1$cohort<=1980]="1976-1980"
NHANES1$cohort.range[NHANES1$cohort>=1981 & NHANES1$cohort<=1985]="1981-1985"


NHANES1$cohort.range[NHANES1$cohort>=1986 & NHANES1$cohort<=1990]="1986-1990"
NHANES1$cohort.range[NHANES1$cohort>=1991 & NHANES1$cohort<=1996]="1991-1996"

#log + 1 transform the PHQ-9
NHANES1$lPHQ9.Total=log(NHANES1$PHQ9.Total+1)

#RPC MOdel
RPC=lm(lPHQ9.Total~ RI+factor(NHANES.year)+factor(cohort.range),data=NHANES1)
#APC model
APC=lm(lPHQ9.Total~ Age+factor(NHANES.year)+factor(cohort.range),data=NHANES1)

#APC model including risk factors
APC2=lm(lPHQ9.Total~ Age+Gender+Race1+Family.PIR+X..of.comorbidities+Social.Support+factor(NHANES.year)+factor(cohort.range),data=NHANES1)

summary(RPC)

##
## Call:
## lm(formula = lPHQ9.Total ~ RI + factor(NHANES.year) + factor(cohort.range),
## data = NHANES1)
##
## Residuals:
## Min 1Q Median 3Q Max
## -2.22655 -0.88294 -0.02845 0.65596 2.54470
##
## Coefficients:
## Estimate Std. Error t value Pr(>|t|)
## (Intercept) -0.4259568 0.0702573 -6.063 1.36e-09 ***
## RI 0.0230374 0.0006882 33.473 < 2e-16 ***
## factor(NHANES.year)2008 0.1088128 0.0184888 5.885 4.02e-09 ***
## factor(NHANES.year)2010 0.0952294 0.0183047 5.202 1.98e-07 ***
## factor(NHANES.year)2012 0.0290017 0.0189181 1.533 0.12528
## factor(NHANES.year)2014 0.0530197 0.0185983 2.851 0.00436 **
## factor(NHANES.year)2016 0.0527404 0.0190207 2.773 0.00556 **
## factor(cohort.range)1926-1930 -0.1106515 0.0675160 -1.639 0.10125
## factor(cohort.range)1931-1935 0.0184903 0.0649771 0.285 0.77598
## factor(cohort.range)1936-1940 0.0250950 0.0639889 0.392 0.69493
## factor(cohort.range)1941-1945 0.0556348 0.0636357 0.874 0.38198
## factor(cohort.range)1946-1950 0.1656213 0.0632384 2.619 0.00882 **
## factor(cohort.range)1951-1955 0.2800564 0.0632573 4.427 9.58e-06 ***
## factor(cohort.range)1956-1960 0.2885093 0.0631589 4.568 4.95e-06 ***
## factor(cohort.range)1961-1965 0.3413655 0.0631944 5.402 6.65e-08 ***
## factor(cohort.range)1966-1970 0.3280781 0.0633335 5.180 2.23e-07 ***
## factor(cohort.range)1971-1975 0.3151332 0.0633321 4.976 6.53e-07 ***
## factor(cohort.range)1976-1980 0.3740252 0.0633617 5.903 3.61e-09 ***
## factor(cohort.range)1981-1985 0.3666201 0.0633668 5.786 7.30e-09 ***
## factor(cohort.range)1986-1990 0.4571446 0.0633514 7.216 5.49e-13 ***
## factor(cohort.range)1991-1996 0.5004917 0.0679785 7.362 1.86e-13 ***
## ---
## Signif. codes: 0 '***' 0.001 '**' 0.01 '*' 0.05 '.' 0.1 ' ' 1
##
## Residual standard error: 0.8734 on 27475 degrees of freedom
## Multiple R-squared: 0.04643, Adjusted R-squared: 0.04573
## F-statistic: 66.89 on 20 and 27475 DF, p-value: < 2.2e-16

summary(APC)

##
## Call:
## lm(formula = lPHQ9.Total ~ Age + factor(NHANES.year) + factor(cohort.range),
## data = NHANES1)
##
## Residuals:
## Min 1Q Median 3Q Max
## -1.12890 -0.95455 0.00213 0.67553 2.36581
##
## Coefficients:
## Estimate Std. Error t value Pr(>|t|)
## (Intercept) 0.613569 0.325741 1.884 0.0596 .
## Age 0.002834 0.003837 0.739 0.4602
## factor(NHANES.year)2008 0.112783 0.020320 5.550 2.88e-08 ***
## factor(NHANES.year)2010 0.098578 0.023518 4.192 2.78e-05 ***
## factor(NHANES.year)2012 0.025999 0.029964 0.868 0.3856
## factor(NHANES.year)2014 0.064282 0.035455 1.813 0.0698 .
## factor(NHANES.year)2016 0.068470 0.042566 1.609 0.1077
## factor(cohort.range)1926-1930 -0.105820 0.072914 -1.451 0.1467
## factor(cohort.range)1931-1935 0.044478 0.077659 0.573 0.5668
## factor(cohort.range)1936-1940 0.031256 0.087351 0.358 0.7205
## factor(cohort.range)1941-1945 0.049881 0.101735 0.490 0.6239
## factor(cohort.range)1946-1950 0.149244 0.116665 1.279 0.2008
## factor(cohort.range)1951-1955 0.241000 0.133062 1.811 0.0701 .
## factor(cohort.range)1956-1960 0.239445 0.149855 1.598 0.1101
## factor(cohort.range)1961-1965 0.261751 0.167459 1.563 0.1180
## factor(cohort.range)1966-1970 0.236113 0.185541 1.273 0.2032
## factor(cohort.range)1971-1975 0.221354 0.203237 1.089 0.2761
## factor(cohort.range)1976-1980 0.271124 0.221771 1.223 0.2215
## factor(cohort.range)1981-1985 0.255151 0.240029 1.063 0.2878
## factor(cohort.range)1986-1990 0.333538 0.257933 1.293 0.1960
## factor(cohort.range)1991-1996 0.371587 0.277071 1.341 0.1799
## ---
## Signif. codes: 0 '***' 0.001 '**' 0.01 '*' 0.05 '.' 0.1 ' ' 1
##
## Residual standard error: 0.891 on 27475 degrees of freedom
## Multiple R-squared: 0.007562, Adjusted R-squared: 0.006839
## F-statistic: 10.47 on 20 and 27475 DF, p-value: < 2.2e-16

summary(APC2)

##
## Call:
## lm(formula = lPHQ9.Total ~ Age + Gender + Race1 + Family.PIR +
## X..of.comorbidities + Social.Support + factor(NHANES.year) +
## factor(cohort.range), data = NHANES1)
##
## Residuals:
## Min 1Q Median 3Q Max
## -2.90016 -0.74974 -0.03029 0.63478 2.69436
##
## Coefficients:
## Estimate Std. Error t value Pr(>|t|)
## (Intercept) 0.896935 0.307269 2.919 0.003514 **
## Age -0.002680 0.003620 -0.740 0.459083
## GenderFemale 0.214729 0.010214 21.023 < 2e-16 ***
## Race1Hispanic -0.064698 0.013334 -4.852 1.23e-06 ***
## Race1Non-Hispanic Black -0.108756 0.013718 -7.928 2.31e-15 ***
## Race1Other Race -0.080853 0.018966 -4.263 2.02e-05 ***
## Family.PIR -0.080282 0.003325 -24.143 < 2e-16 ***
## X..of.comorbidities 0.195742 0.004983 39.284 < 2e-16 ***
## Social.Supportyes -0.127497 0.011007 -11.583 < 2e-16 ***
## factor(NHANES.year)2008 0.094761 0.019173 4.943 7.76e-07 ***
## factor(NHANES.year)2010 0.082463 0.022196 3.715 0.000203 ***
## factor(NHANES.year)2012 0.016940 0.028368 0.597 0.550416
## factor(NHANES.year)2014 0.051585 0.033488 1.540 0.123470
## factor(NHANES.year)2016 0.051657 0.040210 1.285 0.198913
## factor(cohort.range)1926-1930 -0.090475 0.068745 -1.316 0.188153
## factor(cohort.range)1931-1935 0.036487 0.073254 0.498 0.618426
## factor(cohort.range)1936-1940 0.051882 0.082405 0.630 0.528965
## factor(cohort.range)1941-1945 0.089598 0.095995 0.933 0.350643
## factor(cohort.range)1946-1950 0.213878 0.110096 1.943 0.052068 .
## factor(cohort.range)1951-1955 0.325107 0.125519 2.590 0.009600 **
## factor(cohort.range)1956-1960 0.333721 0.141347 2.361 0.018232 *
## factor(cohort.range)1961-1965 0.359673 0.157932 2.277 0.022771 *
## factor(cohort.range)1966-1970 0.345951 0.174979 1.977 0.048041 *
## factor(cohort.range)1971-1975 0.318554 0.191665 1.662 0.096516 .
## factor(cohort.range)1976-1980 0.346399 0.209118 1.656 0.097637 .
## factor(cohort.range)1981-1985 0.299769 0.226303 1.325 0.185303
## factor(cohort.range)1986-1990 0.305446 0.243150 1.256 0.209052
## factor(cohort.range)1991-1996 0.327323 0.261187 1.253 0.210138
## ---
## Signif. codes: 0 '***' 0.001 '**' 0.01 '*' 0.05 '.' 0.1 ' ' 1
##
## Residual standard error: 0.8399 on 27468 degrees of freedom
## Multiple R-squared: 0.1184, Adjusted R-squared: 0.1176
## F-statistic: 136.7 on 27 and 27468 DF, p-value: < 2.2e-16

#RPC Multilevel Model with fixed effect Risk and random effects for period and cohort
library(lme4)

## Warning: package 'lme4' was built under R version 3.5.3

## Loading required package: Matrix


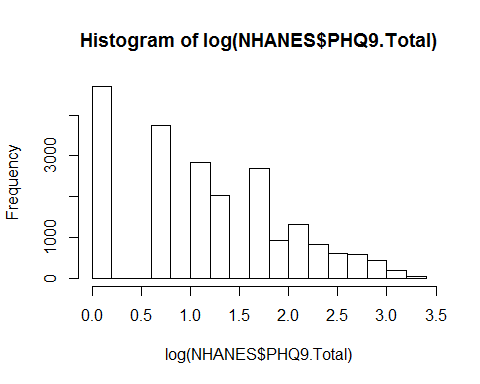


library(lmerTest)

## Warning: package 'lmerTest' was built under R version 3.5.3

##
## Attaching package: 'lmerTest'

## The following object is masked from 'package:lme4':
##
## lmer

## The following object is masked from 'package:stats':
##
## step

RPC_multilevel=lmer(lPHQ9.Total~ RI+(1 | NHANES.year)+(1 | cohort.range),data=NHANES1)
summary(RPC_multilevel)

## Linear mixed model fit by REML. t-tests use Satterthwaite's method [
## lmerModLmerTest]
## Formula: lPHQ9.Total ~ RI + (1 | NHANES.year) + (1 | cohort.range)
## Data: NHANES1
##
## REML criterion at convergence: 70675.3
##
## Scaled residuals:
## Min 1Q Median 3Q Max
## -2.54226 -1.01333 -0.03297 0.75230 2.90569
##
## Random effects:
## Groups Name Variance Std.Dev.
## cohort.range (Intercept) 0.033433 0.18285
## NHANES.year (Intercept) 0.001469 0.03833
## Residual 0.762784 0.87338
## Number of obs: 27496, groups: cohort.range, 15; NHANES.year, 6
##
## Fixed effects:
## Estimate Std. Error df t value Pr(>|t|)
## (Intercept) -1.326e-01 6.058e-02 3.400e+01 -2.19 0.0355 *
## RI 2.287e-02 6.863e-04 2.649e+04 33.32 <2e-16 ***
## ---
## Signif. codes: 0 '***' 0.001 '**' 0.01 '*' 0.05 '.' 0.1 ' ' 1
##
## Correlation of Fixed Effects:
## (Intr)
## RI -0.561

ranef(RPC_multilevel)

## $cohort.range
## (Intercept)
## 1921-1925 -0.21044109
## 1926-1930 -0.32597055
## 1931-1935 -0.20533741
## 1936-1940 -0.19941622
## 1941-1945 -0.16979865
## 1946-1950 -0.06127105
## 1951-1955 0.05112896
## 1956-1960 0.05973821
## 1961-1965 0.11160728
## 1966-1970 0.09838381
## 1971-1975 0.08521625
## 1976-1980 0.14314145
## 1981-1985 0.13562517
## 1986-1990 0.22457311
## 1991-1996 0.26282071
##
## $NHANES.year
## (Intercept)
## 2006 -0.051699398
## 2008 0.046093833
## 2010 0.034376557
## 2012 -0.024171850
## 2014 -0.002365848
## 2016 -0.002233294
##
## with conditional variances for "cohort.range" "NHANES.year"

#APC multilevel model with fixed effect age and age^2 and random effects for period and cohort
APC_multilevel=lmer(lPHQ9.Total~ Age+poly(Age,2)+(1 | NHANES.year)+(1 | cohort.range),data=NHANES1)

## fixed-effect model matrix is rank deficient so dropping 1 column / coefficient

## Warning: Some predictor variables are on very different scales: consider
## rescaling

## Warning: Some predictor variables are on very different scales: consider
## rescaling

summary(APC_multilevel)

## Linear mixed model fit by REML. t-tests use Satterthwaite's method [
## lmerModLmerTest]
## Formula:
## lPHQ9.Total ~ Age + poly(Age, 2) + (1 | NHANES.year) + (1 | cohort.range)
## Data: NHANES1
##
## REML criterion at convergence: 71738.4
##
## Scaled residuals:
## Min 1Q Median 3Q Max
## -1.27231 -1.07253 0.01079 0.75492 2.66537
##
## Random effects:
## Groups Name Variance Std.Dev.
## cohort.range (Intercept) 0.003744 0.06119
## NHANES.year (Intercept) 0.002391 0.04889
## Residual 0.793768 0.89094
## Number of obs: 27496, groups: cohort.range, 15; NHANES.year, 6
##
## Fixed effects:
## Estimate Std. Error df t value Pr(>|t|)
## (Intercept) 1.1361268 0.0491392 11.5207633 23.121 5.16e-11 ***
## Age -0.0026458 0.0008103 9.8927486 -3.265 0.00862 **
## poly(Age, 2)2 2.2377125 1.7868595 46.6748755 1.252 0.21669
## ---
## Signif. codes: 0 '***' 0.001 '**' 0.01 '*' 0.05 '.' 0.1 ' ' 1
##
## Correlation of Fixed Effects:
## (Intr) Age
## Age -0.844
## poly(Ag,2)2 0.066 -0.139
## fit warnings:
## fixed-effect model matrix is rank deficient so dropping 1 column / coefficient
## Some predictor variables are on very different scales: consider rescaling

ranef(APC_multilevel)

## $cohort.range
## (Intercept)
## 1921-1925 -0.008381472
## 1926-1930 -0.111576245
## 1931-1935 -0.008313861
## 1936-1940 -0.034411397
## 1941-1945 -0.034979686
## 1946-1950 0.036399178
## 1951-1955 0.098841648
## 1956-1960 0.075769551
## 1961-1965 0.071372805
## 1966-1970 0.020795753
## 1971-1975 -0.020993011
## 1976-1980 -0.006945604
## 1981-1985 -0.054235250
## 1986-1990 -0.016485679
## 1991-1996 -0.006856730
##
## $NHANES.year
## (Intercept)
## 2006 -0.08468010
## 2008 0.03172117
## 2010 0.02789317
## 2012 -0.02658730
## 2014 0.01869638
## 2016 0.03295669
##
## with conditional variances for "cohort.range" "NHANES.year"

#APC mulitlevel model including risk factors
APC2_multilevel=lmer(lPHQ9.Total~ Age+Gender+Race1+Family.PIR+X..of.comorbidities+Social.Support+poly(Age,2)+(1 | NHANES.year)+(1 | cohort.range),data=NHANES1)

## fixed-effect model matrix is rank deficient so dropping 1 column / coefficient

## Warning: Some predictor variables are on very different scales: consider
## rescaling

## Warning: Some predictor variables are on very different scales: consider
## rescaling

summary(APC2_multilevel)

## Linear mixed model fit by REML. t-tests use Satterthwaite's method [
## lmerModLmerTest]
## Formula:
## lPHQ9.Total ~ Age + Gender + Race1 + Family.PIR + X..of.comorbidities +
## Social.Support + poly(Age, 2) + (1 | NHANES.year) + (1 |
## cohort.range)
## Data: NHANES1
##
## REML criterion at convergence: 68534
##
## Scaled residuals:
## Min 1Q Median 3Q Max
## -3.4507 -0.8936 -0.0368 0.7523 3.2258
##
## Random effects:
## Groups Name Variance Std.Dev.
## cohort.range (Intercept) 0.001880 0.04336
## NHANES.year (Intercept) 0.001338 0.03658
## Residual 0.705468 0.83992
## Number of obs: 27496, groups: cohort.range, 15; NHANES.year, 6
##
## Fixed effects:
## Estimate Std. Error df t value Pr(>|t|)
## (Intercept) 1.447e+00 3.945e-02 1.511e+01 36.667 3.52e-16
## Age -7.689e-03 6.341e-04 1.143e+01 -12.126 7.07e-08
## GenderFemale 2.150e-01 1.021e-02 2.748e+04 21.055 < 2e-16
## Race1Hispanic -6.332e-02 1.331e-02 2.724e+04 -4.756 1.98e-06
## Race1Non-Hispanic Black -1.096e-01 1.371e-02 2.739e+04 -7.997 1.33e-15
## Race1Other Race -8.304e-02 1.894e-02 2.567e+04 -4.385 1.16e-05
## Family.PIR -8.046e-02 3.326e-03 2.748e+04 -24.189 < 2e-16
## X..of.comorbidities 1.961e-01 4.981e-03 2.745e+04 39.370 < 2e-16
## Social.Supportyes -1.305e-01 1.104e-02 2.679e+04 -11.816 < 2e-16
## poly(Age, 2)2 -8.162e+00 1.529e+00 3.260e+01 -5.338 7.05e-06
##
## (Intercept) ***
## Age ***
## GenderFemale ***
## Race1Hispanic ***
## Race1Non-Hispanic Black ***
## Race1Other Race ***
## Family.PIR ***
## X..of.comorbidities ***
## Social.Supportyes ***
## poly(Age, 2)2 ***
## ---
## Signif. codes: 0 '***' 0.001 '**' 0.01 '*' 0.05 '.' 0.1 ' ' 1
##
## Correlation of Fixed Effects:
## (Intr) Age GndrFm Rc1Hsp R1N-HB Rc1OtR Fm.PIR X..f.c Scl.Sp
## Age -0.785
## GenderFemal -0.154 0.009
## Race1Hispnc -0.199 0.037 -0.015
## Rc1Nn-HspnB -0.188 0.031 0.010 0.360
## Race1OthrRc -0.113 0.030 -0.003 0.256 0.243
## Family.PIR -0.197 -0.039 0.014 0.236 0.113 0.011
## X..f.cmrbdt 0.034 -0.223 -0.040 0.099 0.034 0.063 0.126
## Scl.Spprtys -0.120 -0.043 0.105 -0.040 0.127 -0.013 -0.165 0.037
## poly(Ag,2)2 0.015 -0.127 0.012 0.039 0.058 0.036 0.063 -0.021 0.127
## fit warnings:
## fixed-effect model matrix is rank deficient so dropping 1 column / coefficient
## Some predictor variables are on very different scales: consider rescaling

ranef(APC2_multilevel)

## $cohort.range
## (Intercept)
## 1921-1925 0.021861823
## 1926-1930 -0.060359924
## 1931-1935 -0.002855836
## 1936-1940 -0.033092172
## 1941-1945 -0.056055413
## 1946-1950 0.008514445
## 1951-1955 0.067672462
## 1956-1960 0.045576772
## 1961-1965 0.045162732
## 1966-1970 0.017672117
## 1971-1975 -0.014803838
## 1976-1980 0.007080649
## 1981-1985 -0.028728393
## 1986-1990 -0.013423055
## 1991-1996 -0.004222368
##
## $NHANES.year
## (Intercept)
## 2006 -0.05969441
## 2008 0.02454132
## 2010 0.02235650
## 2012 -0.02408946
## 2014 0.01405489
## 2016 0.02283116
##
## with conditional variances for "cohort.range" "NHANES.year"

#continous period and cohort version of analyses

#standardize versions of age, cohort, period and risk
library(standardize)

## Warning: package 'standardize' was built under R version 3.5.3

NHANES1$RI1=scale(NHANES1$RI)
NHANES1$Age1=scale(NHANES1$Age)
NHANES1$cohort1=scale(NHANES1$cohort)
NHANES1$period1=scale(NHANES1$NHANES.year)


#RPC MOdel
RPC_c=lm(lPHQ9.Total~ RI1+period1+cohort1,data=NHANES1)
#APC model
APC_c=lm(lPHQ9.Total~ Age1+period1+cohort1,data=NHANES1)

#APC model including risk factors
APC2_c=lm(lPHQ9.Total~ Age1+Gender+Race1+Family.PIR+X..of.comorbidities+Social.Support+period1+cohort1, data=NHANES1)

summary(RPC_c)

##
## Call:
## lm(formula = lPHQ9.Total ~ RI1 + period1 + cohort1, data = NHANES1)
##
## Residuals:
## Min 1Q Median 3Q Max
## -2.24065 -0.87006 -0.03125 0.65222 2.48896
##
## Coefficients:
## Estimate Std. Error t value Pr(>|t|)
## (Intercept) 1.016043 0.005279 192.486 <2e-16 ***
## RI1 0.201257 0.006034 33.356 <2e-16 ***
## period1 0.004953 0.005348 0.926 0.354
## cohort1 0.139128 0.006090 22.845 <2e-16 ***
## ---
## Signif. codes: 0 '***' 0.001 '**' 0.01 '*' 0.05 '.' 0.1 ' ' 1
##
## Residual standard error: 0.8753 on 27492 degrees of freedom
## Multiple R-squared: 0.04167, Adjusted R-squared: 0.04156
## F-statistic: 398.4 on 3 and 27492 DF, p-value: < 2.2e-16

summary(APC_c)

##
## Call:
## lm(formula = lPHQ9.Total ~ Age1 + period1 + cohort1, data = NHANES1)
##
## Residuals:
## Min 1Q Median 3Q Max
## -1.12155 -0.97527 0.02785 0.65794 2.36668
##
## Coefficients: (1 not defined because of singularities)
## Estimate Std. Error t value Pr(>|t|)
## (Intercept) 1.016043 0.005384 188.709 < 2e-16 ***
## Age1 -0.040425 0.005391 -7.498 6.66e-14 ***
## period1 0.028002 0.005391 5.194 2.07e-07 ***
## cohort1 NA NA NA NA
## ---
## Signif. codes: 0 '***' 0.001 '**' 0.01 '*' 0.05 '.' 0.1 ' ' 1
##
## Residual standard error: 0.8928 on 27493 degrees of freedom
## Multiple R-squared: 0.002881, Adjusted R-squared: 0.002809
## F-statistic: 39.72 on 2 and 27493 DF, p-value: < 2.2e-16

summary(APC2_c)

##
## Call:
## lm(formula = lPHQ9.Total ~ Age1 + Gender + Race1 + Family.PIR +
## X..of.comorbidities + Social.Support + period1 + cohort1,
## data = NHANES1)
##
## Residuals:
## Min 1Q Median 3Q Max
## -2.85099 -0.74641 -0.03371 0.63804 2.61301
##
## Coefficients: (1 not defined because of singularities)
## Estimate Std. Error t value Pr(>|t|)
## (Intercept) 1.041087 0.015219 68.407 < 2e-16 ***
## Age1 -0.137631 0.005824 -23.632 < 2e-16 ***
## GenderFemale 0.218420 0.010249 21.312 < 2e-16 ***
## Race1Hispanic -0.045548 0.013239 -3.440 0.000582 ***
## Race1Non-Hispanic Black -0.094861 0.013631 -6.959 3.50e-12 ***
## Race1Other Race -0.084473 0.018949 -4.458 8.31e-06 ***
## Family.PIR -0.075161 0.003314 -22.678 < 2e-16 ***
## X..of.comorbidities 0.193908 0.004991 38.855 < 2e-16 ***
## Social.Supportyes -0.105446 0.010789 -9.773 < 2e-16 ***
## period1 0.024622 0.005172 4.760 1.94e-06 ***
## cohort1 NA NA NA NA
## ---
## Signif. codes: 0 '***' 0.001 '**' 0.01 '*' 0.05 '.' 0.1 ' ' 1
##
## Residual standard error: 0.8438 on 27486 degrees of freedom
## Multiple R-squared: 0.1096, Adjusted R-squared: 0.1093
## F-statistic: 376 on 9 and 27486 DF, p-value: < 2.2e-16

#using every level of cohort

#RPC Model
RPC_cohort=lm(lPHQ9.Total~ RI+factor(NHANES.year)+factor(cohort),data=NHANES1)
#APC model
APC_cohort=lm(lPHQ9.Total~ Age+factor(NHANES.year)+factor(cohort),data=NHANES1)

#APC model including risk factors
APC2_cohort=lm(lPHQ9.Total~ Age+Gender+Race1+Family.PIR+X..of.comorbidities+Social.Support+factor(NHANES.year)+factor(cohort),data=NHANES1)

summary(RPC_cohort)

##
## Call:
## lm(formula = lPHQ9.Total ~ RI + factor(NHANES.year) + factor(cohort),
## data = NHANES1)
##
## Residuals:
## Min 1Q Median 3Q Max
## -2.28326 -0.87087 -0.03442 0.65497 2.56880
##
## Coefficients:
## Estimate Std. Error t value Pr(>|t|)
## (Intercept) -0.3957597 0.0981097 -4.034 5.50e-05 ***
## RI 0.0230429 0.0006891 33.437 < 2e-16 ***
## factor(NHANES.year)2008 0.1089751 0.0187442 5.814 6.17e-09 ***
## factor(NHANES.year)2010 0.1014247 0.0186734 5.432 5.64e-08 ***
## factor(NHANES.year)2012 0.0319673 0.0192532 1.660 0.096853 .
## factor(NHANES.year)2014 0.0491886 0.0189617 2.594 0.009489 **
## factor(NHANES.year)2016 0.0488680 0.0193470 2.526 0.011547 *
## factor(cohort)1922 -0.0936839 0.2069318 -0.453 0.650748
## factor(cohort)1923 -0.0158045 0.1810110 -0.087 0.930424
## factor(cohort)1924 0.1008930 0.1810133 0.557 0.577273
## factor(cohort)1925 -0.1727931 0.1622643 -1.065 0.286937
## factor(cohort)1926 -0.0540854 0.1768572 -0.306 0.759749
## factor(cohort)1927 0.1998047 0.1998372 1.000 0.317398
## factor(cohort)1928 -0.0886179 0.1043942 -0.849 0.395956
## factor(cohort)1929 -0.1289307 0.1336504 -0.965 0.334711
## factor(cohort)1930 -0.2186733 0.1018843 -2.146 0.031859 *
## factor(cohort)1931 -0.1114547 0.1256670 -0.887 0.375138
## factor(cohort)1932 -0.0457077 0.1021577 -0.447 0.654573
## factor(cohort)1933 -0.1150245 0.1119373 -1.028 0.304156
## factor(cohort)1934 0.0684262 0.1009463 0.678 0.497874
## factor(cohort)1935 0.0144922 0.1087859 0.133 0.894022
## factor(cohort)1936 0.0499222 0.0998749 0.500 0.617187
## factor(cohort)1937 -0.0255852 0.1048755 -0.244 0.807265
## factor(cohort)1938 -0.0993755 0.1051405 -0.945 0.344581
## factor(cohort)1939 0.0390241 0.1045773 0.373 0.709032
## factor(cohort)1940 -0.0291883 0.1034667 -0.282 0.777866
## factor(cohort)1941 -0.0373851 0.1036722 -0.361 0.718395
## factor(cohort)1942 -0.0181338 0.1023711 -0.177 0.859402
## factor(cohort)1943 -0.0171753 0.1020882 -0.168 0.866396
## factor(cohort)1944 0.1125870 0.1012573 1.112 0.266195
## factor(cohort)1945 0.0640701 0.1015250 0.631 0.527995
## factor(cohort)1946 0.0562728 0.1007520 0.559 0.576489
## factor(cohort)1947 0.1589511 0.1006021 1.580 0.114119
## factor(cohort)1948 0.1373600 0.0997880 1.377 0.168672
## factor(cohort)1949 0.1194131 0.1006284 1.187 0.235366
## factor(cohort)1950 0.1954185 0.1002610 1.949 0.051294 .
## factor(cohort)1951 0.2905149 0.1006683 2.886 0.003906 **
## factor(cohort)1952 0.2410003 0.1003430 2.402 0.016323 *
## factor(cohort)1953 0.2324924 0.1009536 2.303 0.021288 *
## factor(cohort)1954 0.2728651 0.1009301 2.704 0.006865 **
## factor(cohort)1955 0.2147687 0.0998308 2.151 0.031459 *
## factor(cohort)1956 0.2334329 0.0998814 2.337 0.019441 *
## factor(cohort)1957 0.3220017 0.0999068 3.223 0.001270 **
## factor(cohort)1958 0.1530109 0.1003291 1.525 0.127248
## factor(cohort)1959 0.2635761 0.1003369 2.627 0.008621 **
## factor(cohort)1960 0.3146620 0.1004197 3.133 0.001729 **
## factor(cohort)1961 0.2272402 0.1000582 2.271 0.023150 *
## factor(cohort)1962 0.4027711 0.0996737 4.041 5.34e-05 ***
## factor(cohort)1963 0.3366284 0.1002292 3.359 0.000784 ***
## factor(cohort)1964 0.2712793 0.0999682 2.714 0.006659 **
## factor(cohort)1965 0.3103483 0.1002006 3.097 0.001955 **
## factor(cohort)1966 0.3690195 0.0999953 3.690 0.000224 ***
## factor(cohort)1967 0.2943226 0.1009847 2.915 0.003565 **
## factor(cohort)1968 0.3107807 0.1004834 3.093 0.001984 **
## factor(cohort)1969 0.2668960 0.1005925 2.653 0.007977 **
## factor(cohort)1970 0.2489571 0.0994027 2.505 0.012267 *
## factor(cohort)1971 0.2915437 0.0994864 2.930 0.003387 **
## factor(cohort)1972 0.2766690 0.0999984 2.767 0.005666 **
## factor(cohort)1973 0.3623523 0.1004702 3.607 0.000311 ***
## factor(cohort)1974 0.2772583 0.1005458 2.758 0.005828 **
## factor(cohort)1975 0.2126243 0.1006534 2.112 0.034658 *
## factor(cohort)1976 0.2771828 0.0999133 2.774 0.005537 **
## factor(cohort)1977 0.3833229 0.1004778 3.815 0.000136 ***
## factor(cohort)1978 0.3416600 0.1005967 3.396 0.000684 ***
## factor(cohort)1979 0.4103959 0.1005630 4.081 4.50e-05 ***
## factor(cohort)1980 0.3126344 0.0998491 3.131 0.001744 **
## factor(cohort)1981 0.3047646 0.0995168 3.062 0.002197 **
## factor(cohort)1982 0.3400955 0.0999114 3.404 0.000665 ***
## factor(cohort)1983 0.2963082 0.1004595 2.950 0.003185 **
## factor(cohort)1984 0.3939716 0.1001721 3.933 8.41e-05 ***
## factor(cohort)1985 0.3454375 0.1001750 3.448 0.000565 ***
## factor(cohort)1986 0.4859734 0.1007006 4.826 1.40e-06 ***
## factor(cohort)1987 0.4044855 0.0980328 4.126 3.70e-05 ***
## factor(cohort)1988 0.4176050 0.0976017 4.279 1.89e-05 ***
## factor(cohort)1989 0.3816675 0.1040312 3.669 0.000244 ***
## factor(cohort)1990 0.4472882 0.1034904 4.322 1.55e-05 ***
## factor(cohort)1991 0.3802357 0.1065907 3.567 0.000361 ***
## factor(cohort)1992 0.5373421 0.1080902 4.971 6.69e-07 ***
## factor(cohort)1993 0.5089256 0.1147966 4.433 9.32e-06 ***
## factor(cohort)1994 0.4171076 0.1136232 3.671 0.000242 ***
## factor(cohort)1995 0.5430670 0.1599691 3.395 0.000688 ***
## factor(cohort)1996 0.6309411 0.1447495 4.359 1.31e-05 ***
## ---
## Signif. codes: 0 '***' 0.001 '**' 0.01 '*' 0.05 '.' 0.1 ' ' 1
##
## Residual standard error: 0.8728 on 27414 degrees of freedom
## Multiple R-squared: 0.04979, Adjusted R-squared: 0.04699
## F-statistic: 17.74 on 81 and 27414 DF, p-value: < 2.2e-16

summary(APC_cohort)

##
## Call:
## lm(formula = lPHQ9.Total ~ Age + factor(NHANES.year) + factor(cohort),
## data = NHANES1)
##
## Residuals:
## Min 1Q Median 3Q Max
## -1.25602 -0.94372 -0.00388 0.66444 2.39300
##
## Coefficients: (1 not defined because of singularities)
## Estimate Std. Error t value Pr(>|t|)
## (Intercept) 1.203222 0.130669 9.208 < 2e-16 ***
## Age -0.003913 0.001964 -1.992 0.04633 *
## factor(NHANES.year)2008 0.125985 0.019262 6.541 6.24e-11 ***
## factor(NHANES.year)2010 0.130695 0.020103 6.501 8.10e-11 ***
## factor(NHANES.year)2012 0.068745 0.022216 3.094 0.00197 **
## factor(NHANES.year)2014 0.113331 0.024037 4.715 2.43e-06 ***
## factor(NHANES.year)2016 0.131070 0.025888 5.063 4.15e-07 ***
## factor(cohort)1922 -0.036840 0.210567 -0.175 0.86112
## factor(cohort)1923 -0.004631 0.183468 -0.025 0.97986
## factor(cohort)1924 0.053185 0.182900 0.291 0.77122
## factor(cohort)1925 -0.155983 0.162954 -0.957 0.33846
## factor(cohort)1926 -0.132476 0.177509 -0.746 0.45549
## factor(cohort)1927 0.118857 0.200833 0.592 0.55398
## factor(cohort)1928 -0.123330 0.099544 -1.239 0.21538
## factor(cohort)1929 -0.133730 0.130353 -1.026 0.30494
## factor(cohort)1930 -0.250653 0.095021 -2.638 0.00835 **
## factor(cohort)1931 -0.159615 0.120468 -1.325 0.18520
## factor(cohort)1932 -0.079820 0.093689 -0.852 0.39424
## factor(cohort)1933 -0.154031 0.104035 -1.481 0.13873
## factor(cohort)1934 0.026014 0.090775 0.287 0.77444
## factor(cohort)1935 -0.036145 0.099176 -0.364 0.71552
## factor(cohort)1936 -0.022724 0.087929 -0.258 0.79608
## factor(cohort)1937 -0.117602 0.093404 -1.259 0.20801
## factor(cohort)1938 -0.206619 0.093079 -2.220 0.02644 *
## factor(cohort)1939 -0.050218 0.091949 -0.546 0.58496
## factor(cohort)1940 -0.140063 0.090143 -1.554 0.12025
## factor(cohort)1941 -0.162942 0.089895 -1.813 0.06991 .
## factor(cohort)1942 -0.139486 0.087913 -1.587 0.11261
## factor(cohort)1943 -0.165206 0.087166 -1.895 0.05806 .
## factor(cohort)1944 -0.036346 0.085829 -0.423 0.67195
## factor(cohort)1945 -0.087083 0.085863 -1.014 0.31049
## factor(cohort)1946 -0.102626 0.084623 -1.213 0.22524
## factor(cohort)1947 -0.014685 0.084197 -0.174 0.86154
## factor(cohort)1948 -0.051916 0.082984 -0.626 0.53157
## factor(cohort)1949 -0.081704 0.083896 -0.974 0.33013
## factor(cohort)1950 0.002075 0.083360 0.025 0.98014
## factor(cohort)1951 0.057616 0.083804 0.688 0.49176
## factor(cohort)1952 0.008870 0.083482 0.106 0.91538
## factor(cohort)1953 -0.002937 0.084253 -0.035 0.97219
## factor(cohort)1954 0.045491 0.084307 0.540 0.58949
## factor(cohort)1955 -0.054417 0.083092 -0.655 0.51254
## factor(cohort)1956 -0.028620 0.083354 -0.343 0.73133
## factor(cohort)1957 0.053137 0.083645 0.635 0.52526
## factor(cohort)1958 -0.127222 0.084516 -1.505 0.13226
## factor(cohort)1959 -0.024127 0.084801 -0.285 0.77602
## factor(cohort)1960 -0.005848 0.085326 -0.069 0.94536
## factor(cohort)1961 -0.091720 0.085269 -1.076 0.28209
## factor(cohort)1962 0.069796 0.085194 0.819 0.41264
## factor(cohort)1963 -0.022641 0.086387 -0.262 0.79325
## factor(cohort)1964 -0.081783 0.086617 -0.944 0.34508
## factor(cohort)1965 -0.065680 0.087439 -0.751 0.45257
## factor(cohort)1966 0.003601 0.087952 0.041 0.96734
## factor(cohort)1967 -0.093379 0.089651 -1.042 0.29761
## factor(cohort)1968 -0.087028 0.089762 -0.970 0.33229
## factor(cohort)1969 -0.143080 0.090591 -1.579 0.11425
## factor(cohort)1970 -0.160643 0.090061 -1.784 0.07448 .
## factor(cohort)1971 -0.114301 0.091031 -1.256 0.20926
## factor(cohort)1972 -0.139694 0.092438 -1.511 0.13075
## factor(cohort)1973 -0.071162 0.093798 -0.759 0.44805
## factor(cohort)1974 -0.163196 0.094767 -1.722 0.08507 .
## factor(cohort)1975 -0.240933 0.095809 -2.515 0.01192 *
## factor(cohort)1976 -0.191884 0.096006 -1.999 0.04565 *
## factor(cohort)1977 -0.071255 0.097772 -0.729 0.46614
## factor(cohort)1978 -0.125950 0.098844 -1.274 0.20259
## factor(cohort)1979 -0.068244 0.099816 -0.684 0.49417
## factor(cohort)1980 -0.178417 0.100150 -1.782 0.07484 .
## factor(cohort)1981 -0.193815 0.100947 -1.920 0.05487 .
## factor(cohort)1982 -0.168438 0.102464 -1.644 0.10021
## factor(cohort)1983 -0.214118 0.104242 -2.054 0.03998 *
## factor(cohort)1984 -0.129553 0.105115 -1.232 0.21778
## factor(cohort)1985 -0.187404 0.106265 -1.764 0.07782 .
## factor(cohort)1986 -0.060032 0.108002 -0.556 0.57832
## factor(cohort)1987 -0.151113 0.107086 -1.411 0.15821
## factor(cohort)1988 -0.145325 0.108002 -1.346 0.17845
## factor(cohort)1989 -0.182250 0.114590 -1.590 0.11175
## factor(cohort)1990 -0.124519 0.115314 -1.080 0.28023
## factor(cohort)1991 -0.212551 0.119302 -1.782 0.07482 .
## factor(cohort)1992 -0.045948 0.122078 -0.376 0.70664
## factor(cohort)1993 -0.093792 0.129176 -0.726 0.46780
## factor(cohort)1994 -0.182419 0.129392 -1.410 0.15861
## factor(cohort)1995 -0.077336 0.173402 -0.446 0.65561
## factor(cohort)1996 NA NA NA NA
## ---
## Signif. codes: 0 '***' 0.001 '**' 0.01 '*' 0.05 '.' 0.1 ' ' 1
##
## Residual standard error: 0.8904 on 27415 degrees of freedom
## Multiple R-squared: 0.01104, Adjusted R-squared: 0.008156
## F-statistic: 3.826 on 80 and 27415 DF, p-value: < 2.2e-16

summary(APC2_cohort)

##
## Call:
## lm(formula = lPHQ9.Total ~ Age + Gender + Race1 + Family.PIR +
## X..of.comorbidities + Social.Support + factor(NHANES.year) +
## factor(cohort), data = NHANES1)
##
## Residuals:
## Min 1Q Median 3Q Max
## -2.94252 -0.74451 -0.02906 0.63197 2.75303
##
## Coefficients: (1 not defined because of singularities)
## Estimate Std. Error t value Pr(>|t|)
## (Intercept) 1.440502 0.123789 11.637 < 2e-16 ***
## Age -0.009195 0.001857 -4.953 7.36e-07 ***
## GenderFemale 0.214994 0.010218 21.041 < 2e-16 ***
## Race1Hispanic -0.063968 0.013345 -4.793 1.65e-06 ***
## Race1Non-Hispanic Black -0.107700 0.013737 -7.840 4.67e-15 ***
## Race1Other Race -0.079751 0.018982 -4.201 2.66e-05 ***
## Family.PIR -0.080119 0.003327 -24.081 < 2e-16 ***
## X..of.comorbidities 0.195968 0.004987 39.296 < 2e-16 ***
## Social.Supportyes -0.127383 0.011037 -11.541 < 2e-16 ***
## factor(NHANES.year)2008 0.107186 0.018176 5.897 3.74e-09 ***
## factor(NHANES.year)2010 0.113506 0.018979 5.980 2.25e-09 ***
## factor(NHANES.year)2012 0.058460 0.021090 2.772 0.00558 **
## factor(NHANES.year)2014 0.098638 0.022739 4.338 1.44e-05 ***
## factor(NHANES.year)2016 0.113689 0.024526 4.635 3.58e-06 ***
## factor(cohort)1922 -0.076695 0.198503 -0.386 0.69923
## factor(cohort)1923 0.044103 0.172953 0.255 0.79872
## factor(cohort)1924 0.211441 0.172450 1.226 0.22017
## factor(cohort)1925 -0.154623 0.153631 -1.006 0.31420
## factor(cohort)1926 -0.023686 0.167373 -0.142 0.88746
## factor(cohort)1927 0.212219 0.189333 1.121 0.26235
## factor(cohort)1928 -0.079331 0.093859 -0.845 0.39800
## factor(cohort)1929 -0.121845 0.122933 -0.991 0.32162
## factor(cohort)1930 -0.211732 0.089605 -2.363 0.01814 *
## factor(cohort)1931 -0.076539 0.113644 -0.673 0.50064
## factor(cohort)1932 -0.073202 0.088366 -0.828 0.40745
## factor(cohort)1933 -0.143426 0.098133 -1.462 0.14388
## factor(cohort)1934 0.047371 0.085631 0.553 0.58013
## factor(cohort)1935 -0.017732 0.093584 -0.189 0.84972
## factor(cohort)1936 -0.005847 0.082938 -0.071 0.94380
## factor(cohort)1937 -0.072843 0.088131 -0.827 0.40851
## factor(cohort)1938 -0.096349 0.087858 -1.097 0.27280
## factor(cohort)1939 -0.028502 0.086795 -0.328 0.74263
## factor(cohort)1940 -0.067542 0.085091 -0.794 0.42734
## factor(cohort)1941 -0.095931 0.084846 -1.131 0.25821
## factor(cohort)1942 -0.074383 0.083021 -0.896 0.37029
## factor(cohort)1943 -0.099226 0.082287 -1.206 0.22788
## factor(cohort)1944 0.041836 0.081049 0.516 0.60574
## factor(cohort)1945 -0.016774 0.081115 -0.207 0.83617
## factor(cohort)1946 -0.022683 0.079976 -0.284 0.77671
## factor(cohort)1947 0.084132 0.079559 1.057 0.29030
## factor(cohort)1948 0.046604 0.078422 0.594 0.55233
## factor(cohort)1949 0.042601 0.079294 0.537 0.59110
## factor(cohort)1950 0.078185 0.078757 0.993 0.32085
## factor(cohort)1951 0.189812 0.079171 2.397 0.01651 *
## factor(cohort)1952 0.121463 0.078864 1.540 0.12353
## factor(cohort)1953 0.100107 0.079571 1.258 0.20837
## factor(cohort)1954 0.124486 0.079622 1.563 0.11796
## factor(cohort)1955 0.094860 0.078493 1.209 0.22686
## factor(cohort)1956 0.099635 0.078743 1.265 0.20577
## factor(cohort)1957 0.167361 0.079008 2.118 0.03416 *
## factor(cohort)1958 0.011633 0.079841 0.146 0.88415
## factor(cohort)1959 0.089908 0.080140 1.122 0.26192
## factor(cohort)1960 0.137652 0.080579 1.708 0.08760 .
## factor(cohort)1961 0.028316 0.080546 0.352 0.72517
## factor(cohort)1962 0.182327 0.080431 2.267 0.02340 *
## factor(cohort)1963 0.122440 0.081583 1.501 0.13342
## factor(cohort)1964 0.053344 0.081835 0.652 0.51451
## factor(cohort)1965 0.085191 0.082586 1.032 0.30229
## factor(cohort)1966 0.129598 0.083086 1.560 0.11882
## factor(cohort)1967 0.054830 0.084651 0.648 0.51717
## factor(cohort)1968 0.069254 0.084791 0.817 0.41407
## factor(cohort)1969 0.032253 0.085600 0.377 0.70634
## factor(cohort)1970 -0.034347 0.085060 -0.404 0.68636
## factor(cohort)1971 0.007929 0.085999 0.092 0.92654
## factor(cohort)1972 -0.016233 0.087317 -0.186 0.85252
## factor(cohort)1973 0.070322 0.088612 0.794 0.42743
## factor(cohort)1974 -0.036369 0.089520 -0.406 0.68455
## factor(cohort)1975 -0.082012 0.090502 -0.906 0.36484
## factor(cohort)1976 -0.058138 0.090660 -0.641 0.52135
## factor(cohort)1977 0.040667 0.092332 0.440 0.65962
## factor(cohort)1978 -0.013499 0.093309 -0.145 0.88497
## factor(cohort)1979 0.034057 0.094240 0.361 0.71781
## factor(cohort)1980 -0.072724 0.094526 -0.769 0.44169
## factor(cohort)1981 -0.088406 0.095297 -0.928 0.35358
## factor(cohort)1982 -0.067103 0.096698 -0.694 0.48772
## factor(cohort)1983 -0.132132 0.098366 -1.343 0.17919
## factor(cohort)1984 -0.065487 0.099149 -0.660 0.50894
## factor(cohort)1985 -0.122842 0.100228 -1.226 0.22035
## factor(cohort)1986 -0.021451 0.101843 -0.211 0.83318
## factor(cohort)1987 -0.139537 0.100956 -1.382 0.16693
## factor(cohort)1988 -0.146800 0.101817 -1.442 0.14937
## factor(cohort)1989 -0.165696 0.108050 -1.534 0.12516
## factor(cohort)1990 -0.122107 0.108717 -1.123 0.26138
## factor(cohort)1991 -0.185430 0.112466 -1.649 0.09921 .
## factor(cohort)1992 -0.072294 0.115086 -0.628 0.52989
## factor(cohort)1993 -0.090765 0.121781 -0.745 0.45609
## factor(cohort)1994 -0.198009 0.121971 -1.623 0.10451
## factor(cohort)1995 -0.117013 0.163469 -0.716 0.47412
## factor(cohort)1996 NA NA NA NA
## ---
## Signif. codes: 0 '***' 0.001 '**' 0.01 '*' 0.05 '.' 0.1 ' ' 1
##
## Residual standard error: 0.8393 on 27408 degrees of freedom
## Multiple R-squared: 0.1215, Adjusted R-squared: 0.1187
## F-statistic: 43.57 on 87 and 27408 DF, p-value: < 2.2e-16

#Figure 3 of "Relationship Between PHQ-9 Total Score and Birth Cohort by Quintiles of Age-Related Risk Using Local Regression Smoothing (N = 27496)""

library(ggplot2)

## Warning: package 'ggplot2' was built under R version 3.5.3

riskQuintileT.valid=c(-Inf,quantile(NHANES1$RI,probs=c(.20,.40,.60,.80)),Inf)
NHANES1$Quintile=cut(NHANES1$RI, riskQuintileT.valid, labels=c("First","Second", "Third", "Fourth","Fifth"))


p <- ggplot(NHANES1, aes(x = NHANES.year, y = PHQ9.Total, fill=Quintile, group=Quintile,linetype=Quintile))

p1=p+ stat_smooth(method = "loess", se=T, formula = y ~ x,size=1.5,
colour="black", alpha=0.65)+
 #scale_colour_grey(start=0.7, end=0.7)+
 #scale_fill_grey(start=0.7, end=0.7)+
ylab("PHQ-9 Total Score") + xlab("Time (Months)")+
theme_classic()+
 theme(axis.text=element_text(size=15),panel.spacing = unit(2, "lines"),
 axis.title=element_text(size=15),
 plot.title = element_text(hjust=0.5,lineheight=1.2, face="bold",size=10),
legend.position="top")+
 theme(legend.title=element_blank())+
 theme(legend.key.width=unit(3,"cm"))+
 scale_linetype_manual(values=c("dotdash", "dotted","solid","dashed","twodash"))

p1


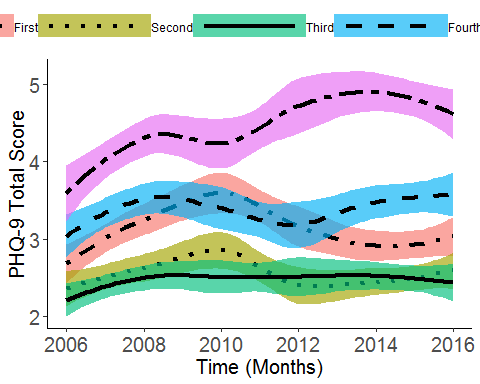

Supplement: S2 File — (ZIP) [file pone.0219399.s002.zip › RPC R Code/NHANES_Analyses_PHQ9_RPC.docx]
