# Supplementary material for: Risk-period-cohort approach for averting identification problems in longitudinal models
Source: PLoS One. 2019 Jul 10;14(7):e0219399. doi: 10.1371/journal.pone.0219399 (PMC6620014; doi:10.1371/journal.pone.0219399)

Risk-Period-Cohort Simulation Study 1

## Simulation Study 1

MC=function(n,slope1,slope2,slope3)
{


#set.seed(114283832)
#n=1000
ID=seq(1:n)

library(truncnorm)

Age <- round(rtruncnorm(n, a=30,b=80, 55, 25 ),digits=0)
Period<-round(rtruncnorm(n,a=1950,b=2010,1980,30),digits=0)


Cohort=Period-Age


X1=rnorm(n,0,1)
X2=rpois(n,1)


age.scale=data.frame(Age,Period,Cohort,X1,X2)

#Y.new=age.scale

 Y.new= data.frame(scale(age.scale))


Y.new$Risk_e=slope1*Y.new$Age+slope2*Y.new$X1+slope3*Y.new$X2+rnorm(n,0,1)
risk.model=lm(Risk_e~Age+X1+X2,data=Y.new)
summary(risk.model)
#age.ratio=abs(risk.model$coefficients[2])/(abs(risk.model$coefficients[2])+abs(risk.model$coefficients[3])+
# abs(risk.model$coefficients[4]))

age.ratio=risk.model$coefficients[2]/(risk.model$coefficients[2]+risk.model$coefficients[3]+
 risk.model$coefficients[4])


risk.model2=lm(Risk_e~X1+X2,data=Y.new)
adj.r1=summary(risk.model)$adj.r.squared
adj.r2=summary(risk.model2)$adj.r.squared
adj.rd=adj.r1-adj.r2
r1=summary(risk.model)$r.squared
r2=summary(risk.model2)$r.squared
rd=r1-r2

Risk=scale(fitted(risk.model))
Y.new$Risk=Risk


cor(Y.new[c(1,2,3,7)])

RA=cor(Y.new$Age,Y.new$Risk)

RP=cor(Y.new$Period,Y.new$Risk)

RC=cor(Y.new$Cohort,Y.new$Risk)

CP=cor(Y.new$Period,Y.new$Cohort)

AP=cor(Y.new$Period,Y.new$Age)

AC=cor(Y.new$Cohort,Y.new$Age)

return(c(slope1,slope2,slope3,RA,RP,RC,CP,AP,AC,adj.r1,adj.r2,adj.rd,r1,r2,rd,age.ratio))


}


#slope=c(-1,-0.5,-0.2,0,0.2,0.5,1)
slope=c(0,.2,.4,.6,.8,1)
l=list(a = slope, b = slope, c = slope)
slope_all=do.call(expand.grid, l)
e=do.call(rbind, replicate(1, slope_all, simplify=FALSE)) # where m is your matrix


MC_a=function(m)
{
slope1=e[m,1]
slope2=e[m,2]
slope3=e[m,3]
x=replicate(1000,MC(10000,slope1,slope2,slope3))
row.mean=apply(x,1,mean)
row.sd=apply(x,1,sd)
row=c(row.mean,row.sd)
return(row)
}

MC_rep=matrix(NA,dim(e)[1],32)
for (i in 1:dim(e)[1])
{
MC_rep[i,]=MC_a(i)
}


MC.rep=data.frame(MC_rep[,c(1:16,19,32)])
colnames(MC.rep)=c("Beta","Alpha1","Alpha2","RA","RP","RC","CP","AP","AC","adj.r1","adj.r2","adj.rd","r1","r2","rd",
 "age.ratio","SD.RA","SD.age.ratio")


MC_rep1=MC.rep[with(MC.rep, order(Beta, Alpha1,Alpha2)),]

MC_rep1$rel.change=((abs(MC_rep1$adj.r1)-abs(MC_rep1$adj.r2))/abs(MC_rep1$adj.r2))*100


#x=MC_rep1[172:343,]
x=MC_rep1

x$rel.change2 <- pmax(pmin(x$rel.change, 300), 0)

library(ggplot2)

## Warning: package 'ggplot2' was built under R version 3.5.3

Sys.setlocale('LC_CTYPE', 'greek')

## [1] "Greek_Greece.1253"

x$Beta=factor(x$Beta,labels=c("1"='\u03b2 = 0',"2"='\u03b2 = 0.2',"3"='\u03b2 = 0.4',
 "4"='\u03b2 = 0.6',"5"='\u03b2 = 0.8',"6"='\u03b2 = 1'))

x$ind=as.factor(as.numeric(x$Beta) >= 4)


x1=x[-1,]


x$ind=as.factor(as.numeric(x$RA) >= 0.85)
x_sub=x[x$ind==TRUE,]

data.frame(x_sub[,c(1:4,16)])

## Beta Alpha1 Alpha2 RA age.ratio
## 2 Î² = 0.2 0.0 0.0 0.9974850 1.0051818
## 3 Î² = 0.4 0.0 0.0 0.9993694 1.0007714
## 39 Î² = 0.4 0.0 0.2 0.8942146 0.6674583
## 9 Î² = 0.4 0.2 0.0 0.8940537 0.6665190
## 4 Î² = 0.6 0.0 0.0 0.9997193 0.9996344
## 40 Î² = 0.6 0.0 0.2 0.9484682 0.7501730
## 10 Î² = 0.6 0.2 0.0 0.9484813 0.7502857
## 46 Î² = 0.6 0.2 0.2 0.9045476 0.6004436
## 5 Î² = 0.8 0.0 0.0 0.9998385 1.0005934
## 41 Î² = 0.8 0.0 0.2 0.9699418 0.8003771
## 77 Î² = 0.8 0.0 0.4 0.8942734 0.6665960
## 11 Î² = 0.8 0.2 0.0 0.9700414 0.8003544
## 47 Î² = 0.8 0.2 0.2 0.9426216 0.6665819
## 83 Î² = 0.8 0.2 0.4 0.8728838 0.5715988
## 17 Î² = 0.8 0.4 0.0 0.8940089 0.6667299
## 53 Î² = 0.8 0.4 0.2 0.8730125 0.5717771
## 6 Î² = 1 0.0 0.0 0.9999013 1.0003184
## 42 Î² = 1 0.0 0.2 0.9805023 0.8336225
## 78 Î² = 1 0.0 0.4 0.9283759 0.7142973
## 114 Î² = 1 0.0 0.6 0.8575958 0.6251611
## 12 Î² = 1 0.2 0.0 0.9804473 0.8332513
## 48 Î² = 1 0.2 0.2 0.9620713 0.7140023
## 84 Î² = 1 0.2 0.4 0.9128926 0.6252211
## 18 Î² = 1 0.4 0.0 0.9284223 0.7145472
## 54 Î² = 1 0.4 0.2 0.9129536 0.6251791
## 90 Î² = 1 0.4 0.4 0.8703412 0.5556974
## 24 Î² = 1 0.6 0.0 0.8574525 0.6250382

x$ind=as.factor(as.numeric(x$SD.age.ratio/1000) >= 0.01)

x$ind2=as.factor(as.numeric(x$RA) <= 0.85 & as.numeric(x$age.ratio>0.55))
x_sub2=x[x$ind2==TRUE,]

data.frame(x_sub2[,c(1:4,16)])

## Beta Alpha1 Alpha2 RA age.ratio
## 1 Î² = 0 0.0 0.0 0.0292172 0.5971186
## 76 Î² = 0.6 0.0 0.4 0.8316133 0.5997861
## 16 Î² = 0.6 0.4 0.0 0.8318873 0.6000385
## 113 Î² = 0.8 0.0 0.6 0.8000360 0.5715089
## 23 Î² = 0.8 0.6 0.0 0.7998704 0.5711950
## 150 Î² = 1 0.0 0.8 0.7807895 0.5554902
## 120 Î² = 1 0.2 0.6 0.8450260 0.5553852
## 60 Î² = 1 0.6 0.2 0.8450483 0.5556974
## 30 Î² = 1 0.8 0.0 0.7805821 0.5554204

x_sub2=x[x$ind==FALSE,]
summary(x_sub2$age_ratio)

## Length Class Mode
## 0 NULL NULL

summary(x_sub$age_ratio)

## Length Class Mode
## 0 NULL NULL

x$Beta_number=as.numeric(x$Beta)
x_not_0=x[x$Beta_number>1,]

p <- ggplot(x, aes(x = age.ratio, y = RA,fill=Beta,color=Beta))

p1=p+ geom_point(size=2,alpha=0.65)+
 facet_wrap(~Beta)+
 scale_x_continuous(breaks=seq(0,1,0.2))+
 geom_hline(aes(yintercept=0.85), colour="#BB0000", linetype="dashed")+
 geom_text(aes(0,0.85,label = 0.85, vjust = -1,hjust=-0.5))+
 geom_hline(aes(yintercept=0.95), colour="blue", linetype="dashed")+
 geom_text(aes(0,0.95,label = 0.95, vjust = -1,hjust=-0.5))+
ylab("Correlation Between Age and Risk") + xlab("Proportionate Age Effect")+
 theme(axis.text=element_text(size=15),
 axis.title=element_text(size=15),
 strip.text = element_text(size=18),
 plot.title = element_text(hjust=0.5,lineheight=1.2, face="bold",size=15)+
 theme(legend.position = "none"))+

 theme_bw( base_size=15)

p1


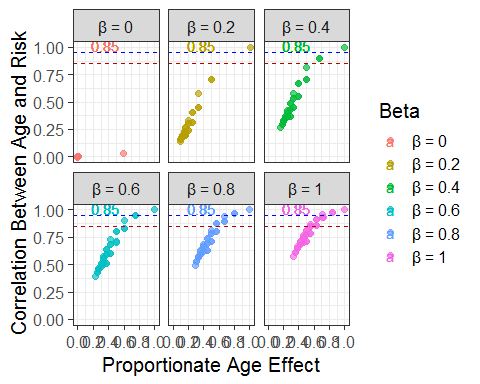

Supplement: S2 File — (ZIP) [file pone.0219399.s002.zip › RPC R Code/RPC Simulation Study 1.docx]
