# Supplementary material for: Risk-period-cohort approach for averting identification problems in longitudinal models
Source: PLoS One. 2019 Jul 10;14(7):e0219399. doi: 10.1371/journal.pone.0219399 (PMC6620014; doi:10.1371/journal.pone.0219399)
Supplement: S2 File — (ZIP) [file pone.0219399.s002.zip › RPC R Code/RPC Simulation Study 2.docx]

Risk-Period-Cohort Simulation Study 2

## RPC Simulation Study 2

MC=function(n,slope1,slope2,slope3,slope4)
{


#e=sample(c(-1,-0.5,-0.2,0,0.2,0.5,1),4)
#slope1=e[1]
#slope2=e[2]
#slope3=e[3]
#slope4=e[4]


#set.seed(114283832)
#n=1000
ID=seq(1:n)

library(truncnorm)

Age <- round(rtruncnorm(n, a=30,b=80, 55, 25 ),digits=0)
Period<-round(rtruncnorm(n,a=1950,b=2010,1980,30),digits=0)


Cohort=Period-Age


X1=rnorm(n,0,1)
X2=rpois(n,1)


age.scale=data.frame(Age,Period,Cohort,X1,X2)
#Y.new=age.scale
Y.new=data.frame(scale(age.scale))


Y.new$Risk_e=slope1*Y.new$Age+slope2*Y.new$X1+slope3*Y.new$X2+rnorm(n,0,1)
risk.model=lm(Risk_e~Age+X1+X2,data=Y.new)
summary(risk.model)

Risk=scale(fitted(risk.model))
Y.new$Risk=Risk

Y.new$APC=slope1*Y.new$Age+slope2*Y.new$X1+slope3*Y.new$X2+slope4*Y.new$Cohort+rnorm(n,0,1)
APC.model=lm(APC~Age+X1+X2+Cohort,data=Y.new)
RPC.model=lm(APC~Risk+Cohort+Period,data=Y.new)
APC=summary(APC.model)
RPC=summary(RPC.model)


APC.results=c(as.vector(APC$coefficients),APC$r.squared,APC$adj.r.squared)

RPC.results=c(as.vector(RPC$coefficients),RPC$r.squared,RPC$adj.r.squared)
BO=cor(Y.new$Risk_e,Y.new$APC)

all.results=c(slope1,slope2,slope3,slope4,APC.results,RPC.results,BO

 )
return(all.results)


}


slope=c(0,0.2,0.4,0.6,0.8,1.0)
l=list(a = slope, b = slope, c = slope, d=slope)
slope_all=do.call(expand.grid, l)
e=do.call(rbind, replicate(1, slope_all, simplify=FALSE)) # where m is your matrix


MC_a=function(m)
{
slope1=e[m,1]
slope2=e[m,2]
slope3=e[m,3]
slope4=e[m,4]
x=replicate(1000,MC(10000,slope1,slope2,slope3,slope4))
row.mean=apply(x,1,mean)
row.sd=apply(x,1,sd)[c(5:9,27:30,45)]
row=c(row.mean,row.sd)
return(row)
}

MC.rep=matrix(NA,dim(e)[1],55)
for (i in 1:dim(e)[1])
{
MC.rep[i,]=MC_a(i)
}


MC_rep_sub=data.frame(MC.rep[,c(1:9,27:30,45,46:55)])
colnames(MC_rep_sub)=c("slope1","slope2","slope3","slope4","Int","Beta","Alpha1","Alpha2","Gamma","Int2","Beta2","Gamma2","Delta","BO","sdI","sdB","sdA1",
"sdA2","sdG","sdI2","sdB2","sdG2","sdD","sdBO")


#MC_rep_c=MC_rep_sub[with(MC_rep_sub, order(slope1, slope2,slope3,slope4)),]
#MC_rep_c$slope <- cumsum(!duplicated(MC_rep_c[1:4]))
#MC_rep1=aggregate(MC_rep_1, list(MC_rep_c$slope), mean)
MC_rep1=MC_rep_sub

MC_rep1$Diff_Beta=MC_rep1$Beta+MC_rep1$Alpha1+MC_rep1$Alpha2-MC_rep1$Beta2
MC_rep1$Diff_Gamma=MC_rep1$Gamma-MC_rep1$Gamma2


MC_rep1$SD_Diff_Gamma=sqrt((MC_rep1$sdG^2)+(MC_rep1$sdG2^2))
MC_rep1$SD_Diff_Beta=sqrt((MC_rep1$sdB^2)+(MC_rep1$sdB2^2))

MC_rep1$CV_Diff_Gamma=MC_rep1$SD_Diff_Gamma/MC_rep1$Diff_Gamma
MC_rep1$CV_Diff_Beta=MC_rep1$SD_Diff_Beta/MC_rep1$Diff_Beta
MC_rep1$CV_Delta=MC_rep1$sdD/MC_rep1$Delta

MC_rep1$Gamma_95L=MC_rep1$Diff_Gamma-(1.96*(MC_rep1$SD_Diff_Gamma/500))
MC_rep1$Gamma_95U=MC_rep1$Diff_Gamma+(1.96*(MC_rep1$SD_Diff_Gamma/500))

MC_rep1$Delta_95L=MC_rep1$Delta-(1.96*(MC_rep1$sdD/500))
MC_rep1$Delta_95U=MC_rep1$Delta+(1.96*(MC_rep1$sdD/500))

MC_rep1$SE.D=MC_rep1$sdD/500

MC_rep1$SE.DG=MC_rep1$SD_Diff_Gamma/500

MC_rep1=MC_rep1[with(MC_rep1, order(Beta, Alpha1,Alpha2,Gamma)),]
MC_rep1$pattern=seq(1:dim(e)[1])

MC_rep2=MC_rep1


MC_high=MC_rep2[abs(MC_rep2$Diff_Gamma)>0.01 | abs(MC_rep2$Delta)>0.01, ]

MC_high1=MC_high[,c("pattern","slope1","slope2","slope3","slope4","Diff_Gamma","Delta","Diff_Beta","BO",
 "sdB","sdG","sdB2","sdG2","sdD","sdBO","SD_Diff_Gamma","SD_Diff_Beta",
 "Gamma_95L","Gamma_95U","Delta_95L","Delta_95U","SE.D","SE.DG")]


library(knitr)

## Warning: package 'knitr' was built under R version 3.5.3

kable(MC_high1[,1:9], digits = 2, align = 'c',
 caption = 'Table. Slope parameter patters with inaccurate cohort or period parameter estimates in risk model')

Table. Slope parameter patters with inaccurate cohort or period parameter estimates in risk model

|  | pattern | slope1 | slope2 | slope3 | slope4 | Diff_Gamma | Delta | Diff_Beta | BO |
| --- | --- | --- | --- | --- | --- | --- | --- | --- | --- |
| 218 | 307 | 0.2 | 0 | 0 | 0.2 | 0.31 | 0.24 | 0.20 | 0.01 |
| 1082 | 312 | 0.2 | 0 | 0 | 1.0 | 0.31 | 0.24 | 0.20 | -0.06 |
| 2 | 322 | 0.2 | 0 | 0 | 0.0 | 0.31 | 0.24 | 0.20 | 0.04 |
| 434 | 331 | 0.2 | 0 | 0 | 0.4 | 0.27 | 0.21 | 0.17 | -0.01 |
| 866 | 355 | 0.2 | 0 | 0 | 0.8 | 0.31 | 0.24 | 0.20 | -0.05 |
| 650 | 424 | 0.2 | 0 | 0 | 0.6 | 0.32 | 0.25 | 0.21 | -0.03 |
| 435 | 460 | 0.4 | 0 | 0 | 0.4 | 0.68 | 0.52 | 0.43 | 0.05 |
| 3 | 463 | 0.4 | 0 | 0 | 0.0 | 0.55 | 0.42 | 0.35 | 0.14 |
| 219 | 530 | 0.4 | 0 | 0 | 0.2 | 0.65 | 0.50 | 0.42 | 0.10 |
| 651 | 539 | 0.4 | 0 | 0 | 0.6 | 0.61 | 0.46 | 0.39 | 0.01 |
| 1083 | 572 | 0.4 | 0 | 0 | 1.0 | 0.73 | 0.56 | 0.47 | -0.07 |
| 867 | 590 | 0.4 | 0 | 0 | 0.8 | 0.64 | 0.49 | 0.41 | -0.04 |
| 436 | 670 | 0.6 | 0 | 0 | 0.4 | 0.76 | 0.58 | 0.49 | 0.16 |
| 1084 | 712 | 0.6 | 0 | 0 | 1.0 | 1.04 | 0.80 | 0.67 | -0.02 |
| 868 | 737 | 0.6 | 0 | 0 | 0.8 | 0.93 | 0.72 | 0.60 | 0.04 |
| 4 | 758 | 0.6 | 0 | 0 | 0.0 | 0.90 | 0.69 | 0.57 | 0.26 |
| 220 | 769 | 0.6 | 0 | 0 | 0.2 | 0.98 | 0.75 | 0.63 | 0.22 |
| 652 | 778 | 0.6 | 0 | 0 | 0.6 | 0.92 | 0.71 | 0.59 | 0.10 |
| 653 | 887 | 0.8 | 0 | 0 | 0.6 | 1.19 | 0.92 | 0.76 | 0.22 |
| 869 | 945 | 0.8 | 0 | 0 | 0.8 | 1.19 | 0.91 | 0.76 | 0.15 |
| 221 | 958 | 0.8 | 0 | 0 | 0.2 | 1.15 | 0.89 | 0.74 | 0.35 |
| 1085 | 983 | 0.8 | 0 | 0 | 1.0 | 1.18 | 0.90 | 0.75 | 0.08 |
| 437 | 1011 | 0.8 | 0 | 0 | 0.4 | 1.26 | 0.97 | 0.81 | 0.29 |
| 5 | 1019 | 0.8 | 0 | 0 | 0.0 | 1.17 | 0.90 | 0.75 | 0.39 |
| 222 | 1086 | 1.0 | 0 | 0 | 0.2 | 1.66 | 1.28 | 1.06 | 0.46 |
| 1086 | 1146 | 1.0 | 0 | 0 | 1.0 | 1.66 | 1.27 | 1.06 | 0.19 |
| 870 | 1168 | 1.0 | 0 | 0 | 0.8 | 1.43 | 1.10 | 0.91 | 0.27 |
| 6 | 1192 | 1.0 | 0 | 0 | 0.0 | 1.67 | 1.28 | 1.07 | 0.50 |
| 438 | 1199 | 1.0 | 0 | 0 | 0.4 | 1.59 | 1.22 | 1.01 | 0.41 |
| 654 | 1275 | 1.0 | 0 | 0 | 0.6 | 1.37 | 1.05 | 0.87 | 0.34 |

#kable(MC_high1[,18:23], digits = 4, align = 'c',
 # caption = 'Table. CIs')
